# Process Safety Assessment of the Iron-Catalyzed 1,2-*cis*-Selective Glycal Aminoglycosylation

**DOI:** 10.1021/acs.oprd.6c00069

**Published:** 2026-05-12

**Authors:** Dakang Zhang, Nicola Colombo, Zixiang Jiang, Davide Pirola, Marino Nebuloni, Hao Xu

**Affiliations:** † Department of Chemistry, 8244 Brandeis University, 415 South Street, Waltham, Massachusetts 02453, United States; ‡ Redox Laboratory, Viale G.B. Stucchi 62/26, Monza, Monza and Brianza 20900, Italy

**Keywords:** catalysis, glycosylation, iron, oligosaccharides, process safety assessment, 1,2-cis-selective

## Abstract

Complex glycans are directly involved in numerous disease processes, and these structural motifs are increasingly incorporated in pharmaceuticals and biological probes. Therefore, the development of highly stereoselective and broadly effective glycosylation processes that are safe and readily scalable has been of great value in both academia and industry. We report herein a process safety assessment of an iron-catalyzed, entirely 1,2-*cis*-selective, glycosylation process for the synthesis of a broad range of biologically important 1,2-*cis*-aminoglycosides. Differential scanning calorimetry analysis of the corresponding catalyst, amination reagents, and an array of representative 1,2-*cis*-aminoglycosides revealed that all of them are thermally stable at temperatures at least 100 °C above the glycosylation temperature. Accelerating rate calorimetry analysis of the amination reagents confirmed the high exothermic decomposition temperatures of these amination reagents and concluded that they present modest maximum self-heat rates and are thereby safe for storage and use in bulk. Drop weight test of the amination reagents suggested that they are impact-stable. Guided by this assessment, we have developed a multigram-scale synthesis of stereochemically pure Tn antigen via the iron-catalyzed 1,2-*cis*-selective glycal aminoglycosylation.

## Graphical abstract

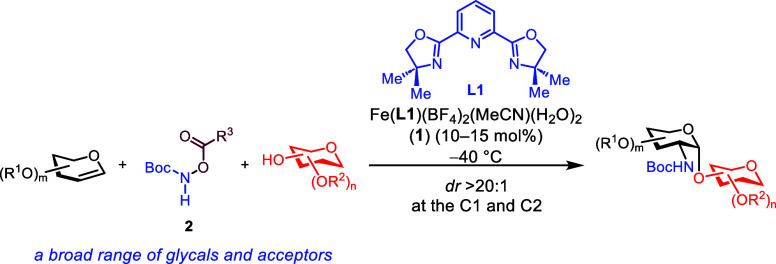

## Introduction

Complex carbohydrates are directly involved in numerous physiological and disease processes, so these structural motifs are increasingly incorporated in pharmaceuticals and biological probes (Figure 1).^1–4^ Over the past century, many powerful chemical glycosylation methods have been invented,^5–11^ but one major hurdle still remains: the stereoselectivity in glycosylation often varies with substrates, so it is difficult to develop a highly stereoselective glycosylation method that is also broadly effective. In particular, there is no general solution for highly 1,2-*cis*-selective glycosylation.^12^

**Figure 1:**
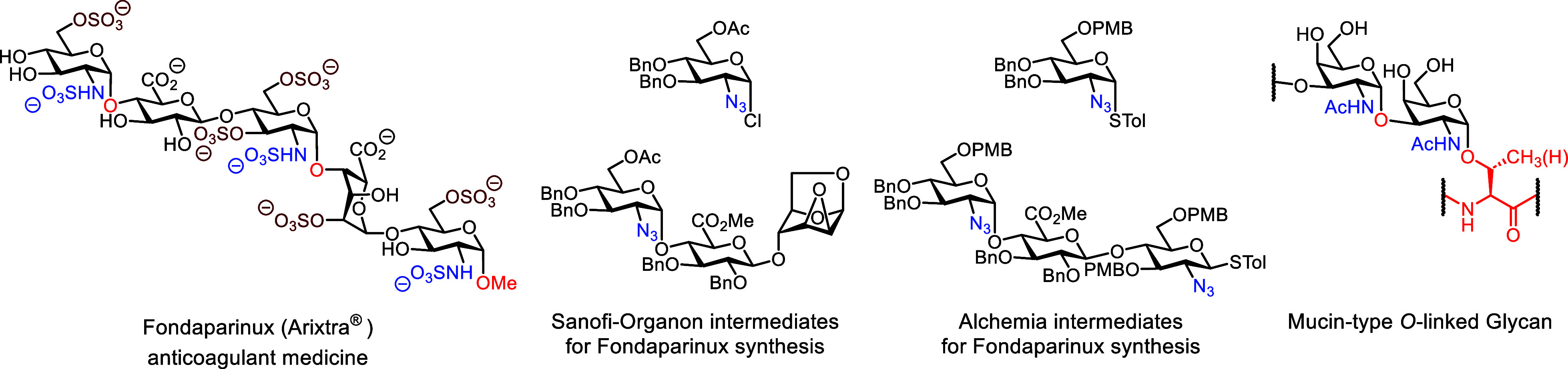
Pharmaceuticals and biologically important complex glycans with 1,2-*cis*-2-aminoglycosidic linkages.

1,2-*cis*-2-Aminoglycosidic linkages are present in many pharmaceuticals and biologically important complex glycans, including anticoagulant heparan sulfate^13^ and Fondaparinux,^1^ aminoglycoside antibiotics, and *O*-linked glycopeptides. Unlike 1,2-*trans*-2-aminoglycosides which can be prepared by established methods,^14–21^ it is still challenging to reliably form 1,2-*cis*-2-aminoglycosidic linkages in high stereoselectivity. The existing *cis*-selective methods leverage non-neighboring-participating groups, particularly the azido group, to enhance the 1,2-*cis*-selectivity (Figures 1 and 2A);^22–32^ however, these methods are most effective for certain types of substrates with specific substituents or conformations. Structural variations in either glycosyl donors or glycosyl acceptors often compromise the high 1,2-*cis*-selectivity. In addition, organic azides intermediates, especially those with low molecular weight, may present potential safety concerns for their handling,^33^ which adds to the difficulty for process development of large-scale 1,2-*cis*-selective aminoglycosylation.

**Figure 2:**
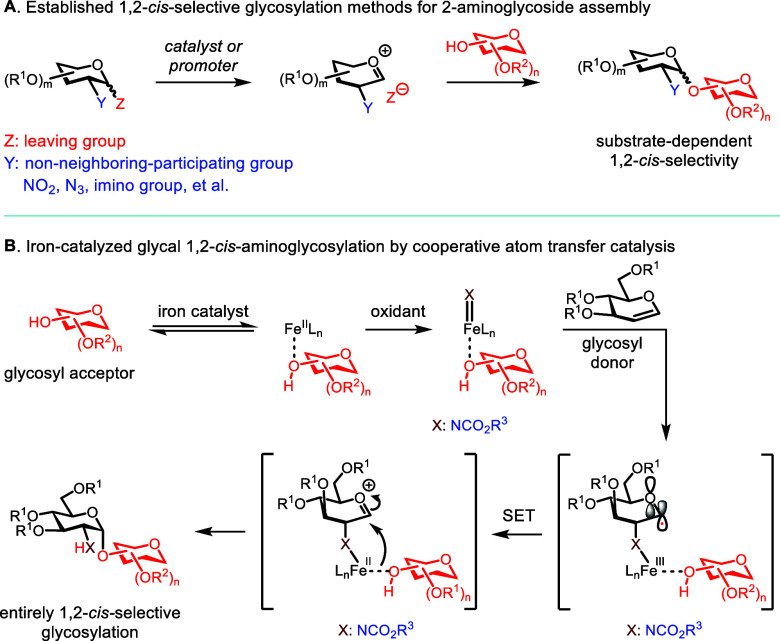
(A) Established glycosylation methods for the synthesis of 1,2-*cis*-aminoglycosides. (B) Iron-catalyzed glycal 1,2-*cis*-aminoglycosylation by cooperative atom transfer catalysis.

Inspired by our research program of the iron-catalyzed selective nitrogen atom transfer for olefin difunctionalization,^34–37^ we envisioned a unique approach that would lead to exclusively 1,2-*cis*-selective aminoglycosylation (Figure 2B). We hypothesized that an iron catalyst could activate a glycosyl acceptor and an amination reagent and subsequently transfer both moieties to a glycal that is otherwise unreactive in the absence of the catalyst. As both the nitrogen atom transfer step and the glycosyl-acceptor transfer step would be directed by the iron catalyst, we predict that the amido group and the catalyst-activated glycosyl acceptor could be cooperatively delivered to a glycal in an exclusively *cis*-selective manner. Distinct from existing glycosylation methods, the unactivated glycosyl acceptor is unlikely to compete for the same reactive intermediate, which should minimize or even eliminate stereochemical erosion from the nonstereoselective pathways.

Directly building upon a pair of iron-catalyzed olefin aminohydroxylation reactions discovered in our lab in 2013 ^34^ and 2014 ^35^ (Figure 3A), we have recently developed an iron-catalyzed 1,2-*cis*-selective glycal aminoglycosylation method (Figure 3B).^38^ A readily available iron catalyst. Fe­(**L1**)­(BF_4_)_2_(MeCN)­(H_2_O)_2_ **1**,^37^ is uniquely effective in promoting the entire 1,2-*cis*-selective aminoglycosylation between a bench-stable glycal, a glycosyl acceptor, and an amination reagent (Figure 3B). Catalyst **1** facilitates facile cleavage of the N–O bond of acyloxy carbamate **2** and transfers both the carbamate fragment of **2** and the glycosyl acceptor to the glycal in an exclusively *cis*-selective manner from the α-face, affording the desired aminoglycoside (d*r* > 20:1). This 1,2-*cis*-selective method is effective for a broad range of glycosyl donors and acceptors (Figure 4). Electron-rich glycals (with 3 electron-donating substituents) are the most reactive substrates, and they are connected with primary acceptors to afford 1,2-*cis*-aminoglycosides using a variety of amination reagents (**2a**–**2c**). However, sterically more hindered amination reagents (**2b**–**2c**) are necessary for glycosylation of these donors with secondary acceptors to minimize the competing *cis*-aminoacyloxylation.^39^ Tri-*O*-acyl glucals are not suitable glycosyl donors; however, just switching one of the *O*-acyl groups to an *O*-silyl group is sufficient to turn on the reactivity. With these substrates, glycosylation with **2a** is effective for both primary and secondary acceptors, while reactions with **2b**–**2c** provide a low conversion. Compared with other carbamate protecting groups, the *N*-Boc activating group is optimal for the high yielding 1,2-*cis*-selective aminoglycosylation, and the *N*-unprotected hydroxylamine derivatives are not compatible with this method.^38^ This iron-catalyzed glycosylation method plays a pivotal role in highly stereoselective synthesis of biologically important Tn antigens^40–42^ and heparan sulfate oligosaccharides^43^ (Figure 3B).

**Figure 3:**
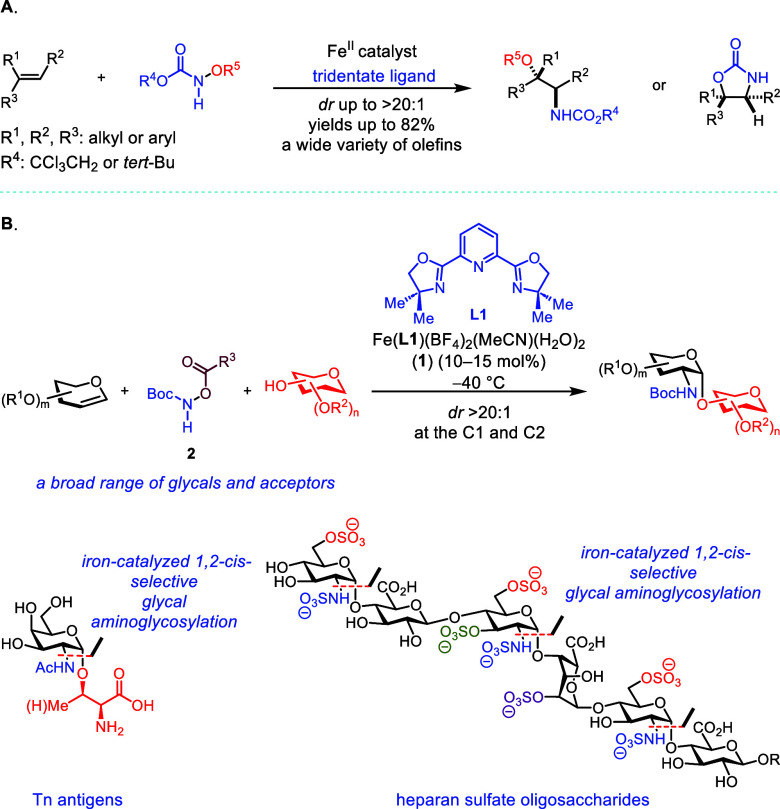
Iron-catalyzed selective nitrogen atom transfer reactions with functionalized hydroxylamines. (A) Iron-catalyzed aminohydroxylation of unfunctionalized olefins. (B) Iron-catalyzed glycal 1,2-*cis*-aminoglycosylation and its application in the highly stereoselective synthesis of Tn antigens and heparan sulfate oligosaccharides.

**Figure 4:**
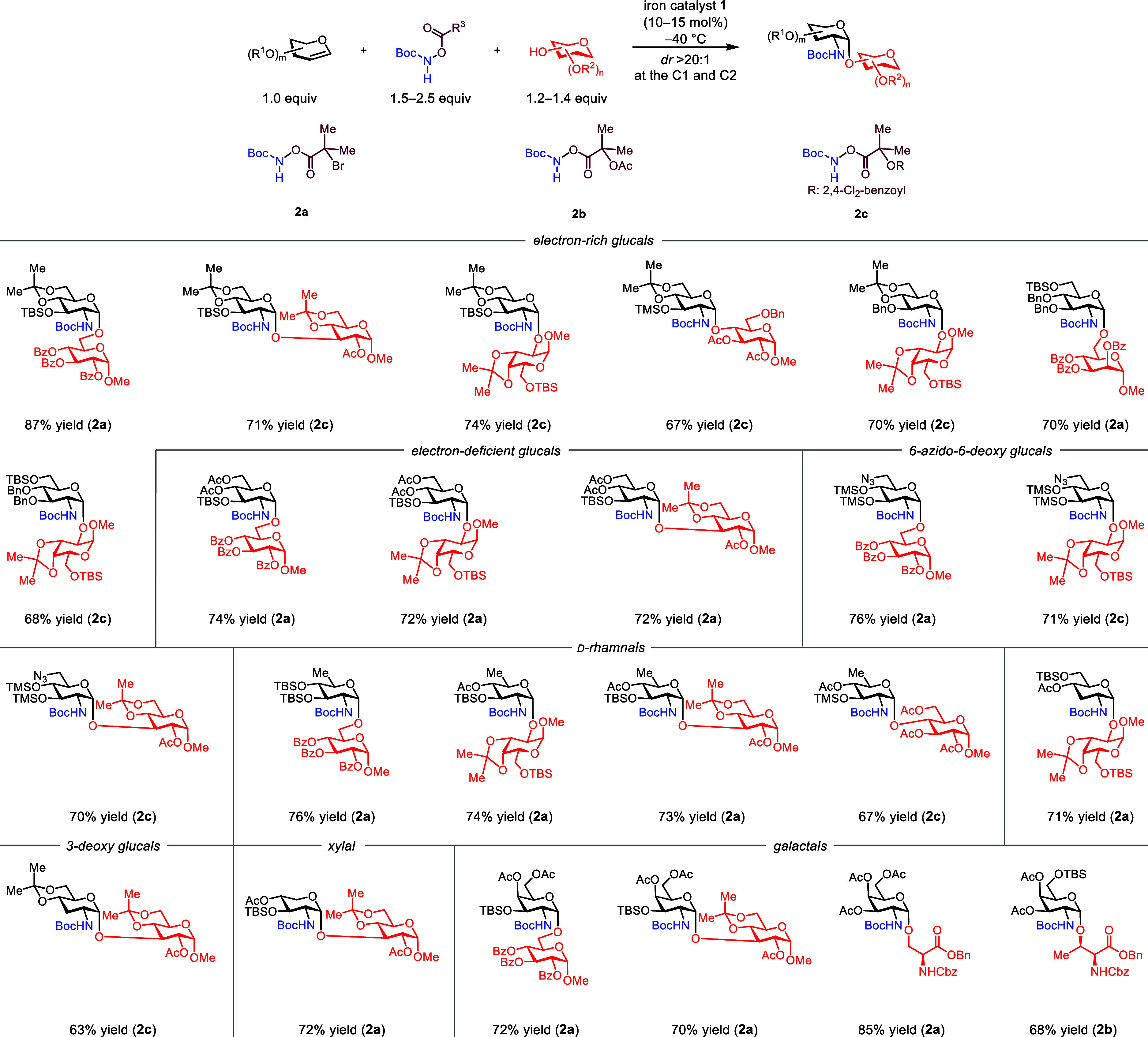
Substrate scope for the iron-catalyzed glycal 1,2-*cis*-aminoglycosylation. All yields are isolated yields.

The iron catalyst **1** capitalizes on the high-energy N–O bond in acyloxy carbamate **2** to drive the glycosylation under cryogenic conditions (−40 °C); however, it is known that thermal hazards could be present in molecules with weak N–O bonds,^44^ which may present potential safety concerns for their handling, with regard to their thermo- and mechanical impact stabilities. In order to explore the feasibility of this iron-catalyzed glycosylation on an industrial scale,^45^ it is imperative to carry out chemical hazard assessment of this reaction. A reactive chemical hazard assessment refers to the identification and possibly quantification of dangerous energy release scenarios for a chemical process of interest. Herein, we describe our chemical safety studies and potential hazard assessment of the iron-catalyzed 1,2-*cis*-selective glycosylation, using differential scanning calorimetry (DSC),^46^ accelerating rate calorimetry (ARC),^47^ and drop weight test (DWT) analysis.

## Results and Discussion

We selected the iron-catalyzed glycosylation of primary and secondary acceptors (**4** and **6**) with glycal **3** and amination reagents **2a**/**2b** as the model reactions for the safety analysis. Kinetic studies of the glycosylation of primary acceptor **4** with electron-rich glycal **3** and amination reagent **2a** (eq 1 in Scheme 1) suggested that its initial rate has a first-order dependence on the iron catalyst, an inverse first-order dependence on acceptor **4**, and a zero-order dependence on both amination reagent **2a** and glycal **3**.^48^ Kinetic studies further revealed that the initial rate for glycosylation of secondary acceptor **6** with amination reagent **2b** and glycal **3** (eq 2 in Scheme 1) has a first-order dependence on the iron catalyst but a zero-order dependence on each of acceptor **6**, glycal **3**, and amination reagent **2b**.^48^ The kinetic study and other mechanistic studies^49^ corroborated a proposed mechanistic working hypothesis for cooperative atom transfer catalysis.^38^

**Scheme 1:**
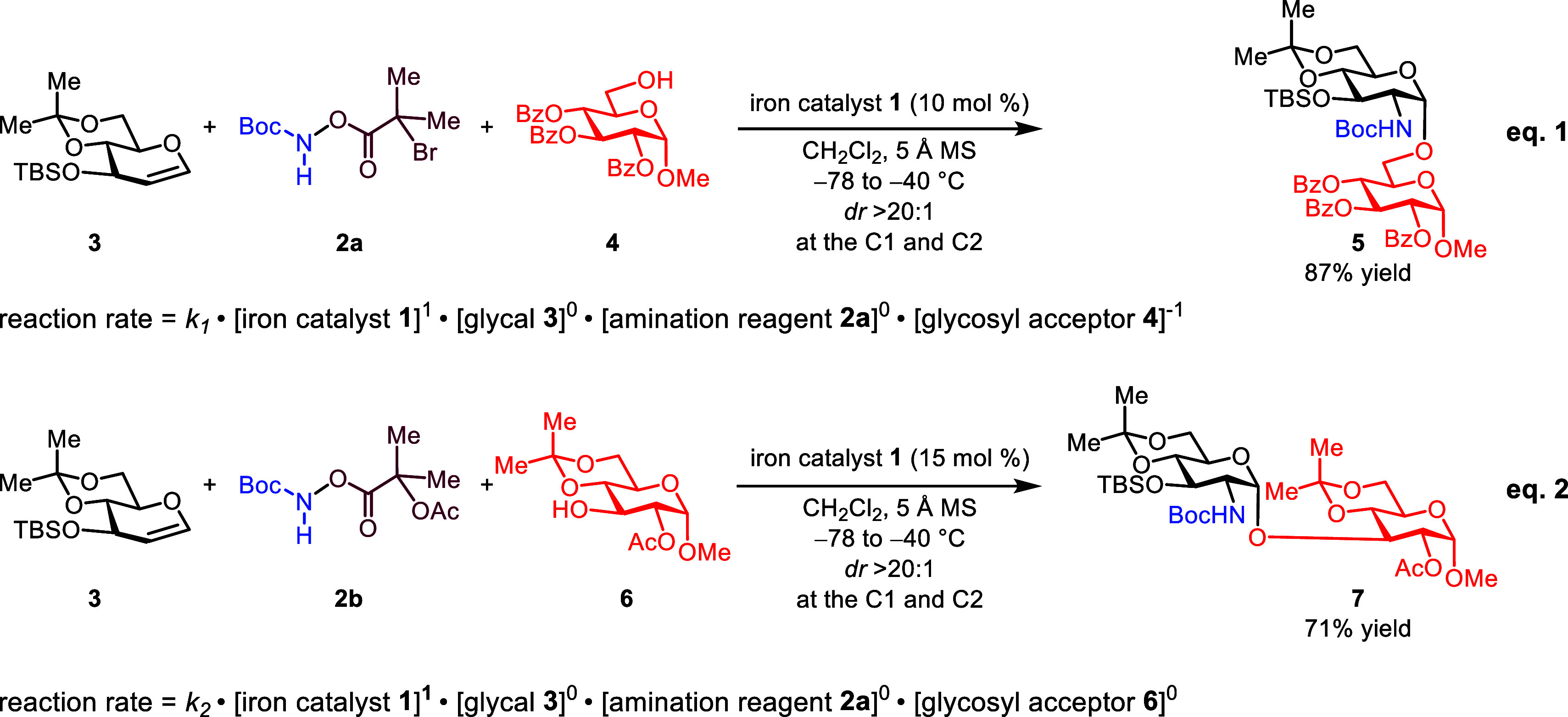
Iron-Catalyzed Glycal 1,2-*cis*-Aminoglycosylation of **4** and **6**

As our first step to explore the feasibility of applying this reaction on an industrial scale, we conducted a series of thermal stability tests using DSC and ARC (Figures 5–8, and S14).

**Figure 5:**
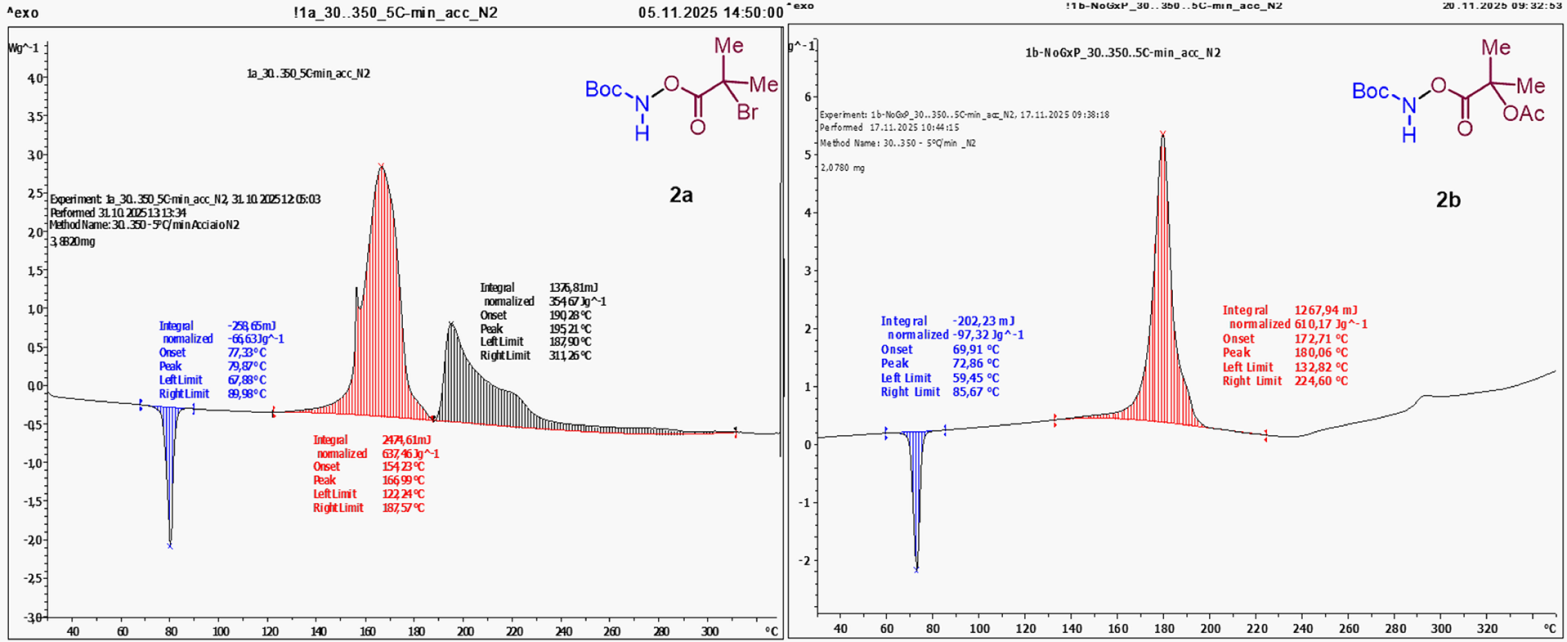
DSC analysis of the amination reagents **2a** (left) and **2b** (right) in the iron-catalyzed glycal 1,2-*cis*-aminoglycosylation.

**Figure 6:**
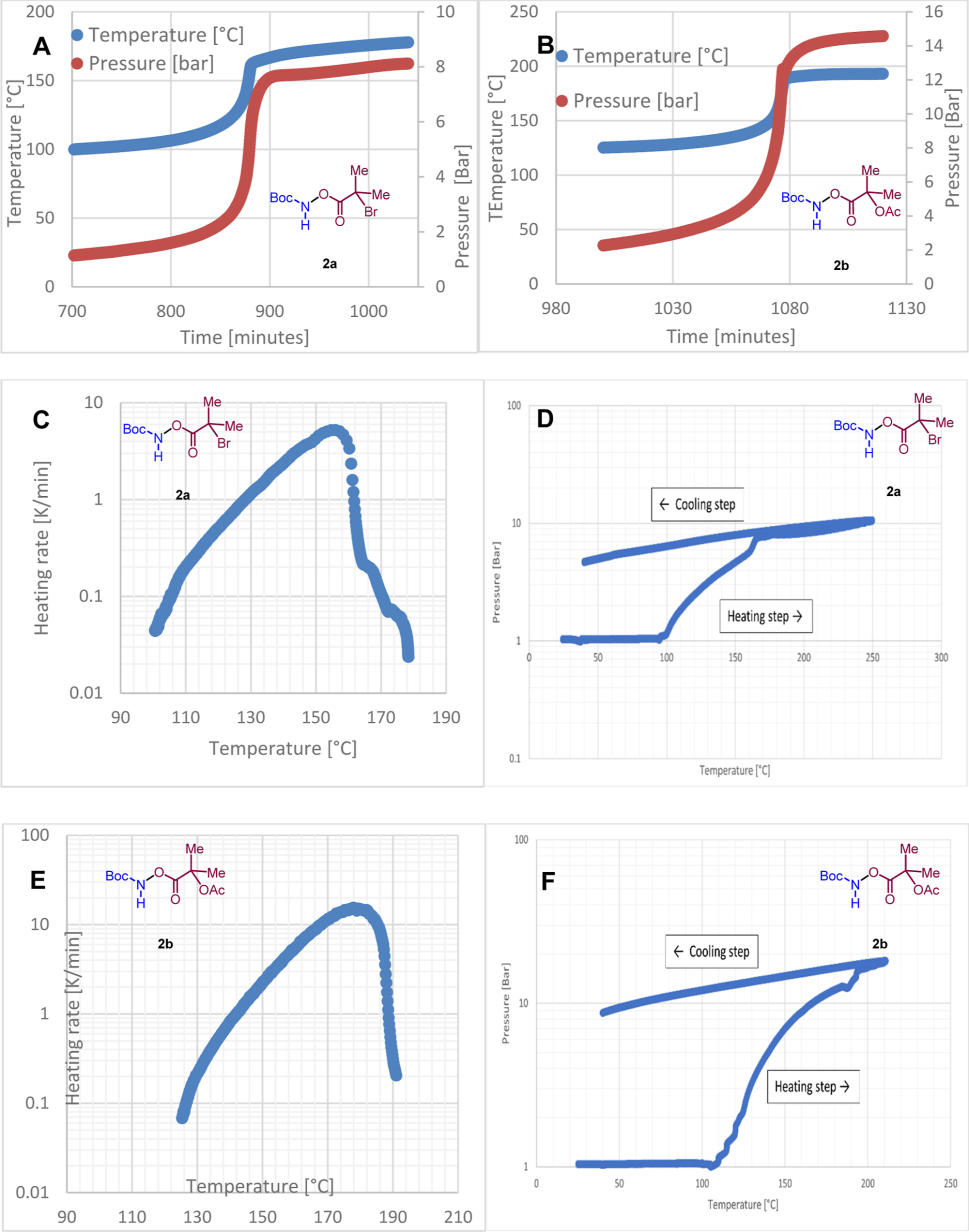
ARC analysis of the amination reagents **2a**/**2b**. (A,B) ARC plots of **2a**/**2b** for temperature and pressure vs time. (C,D) ARC for **2a**. (E,F) ARC for **2b**.

**Figure 7:**
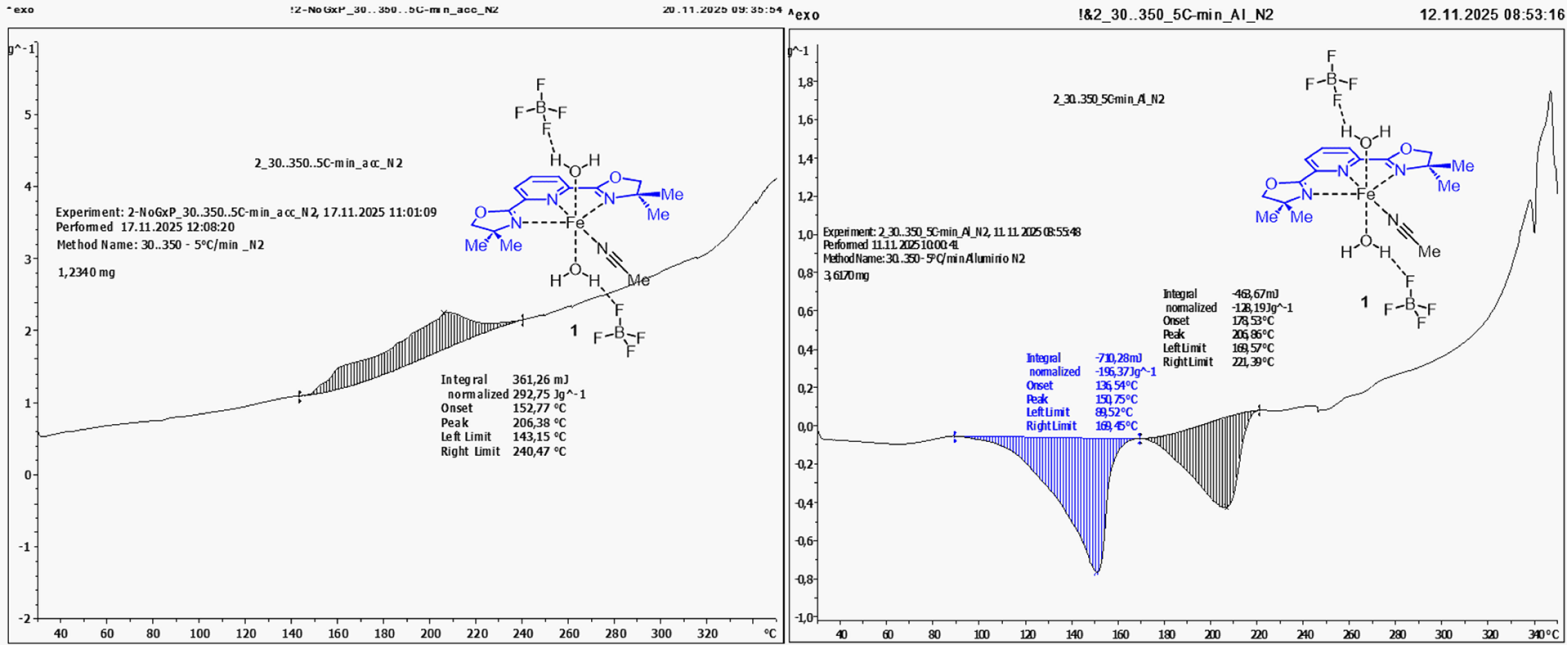
DSC analysis of the iron catalyst **1** in stainless-steel sealed pan (left) and in pierced aluminum pan (right).

**Figure 8:**
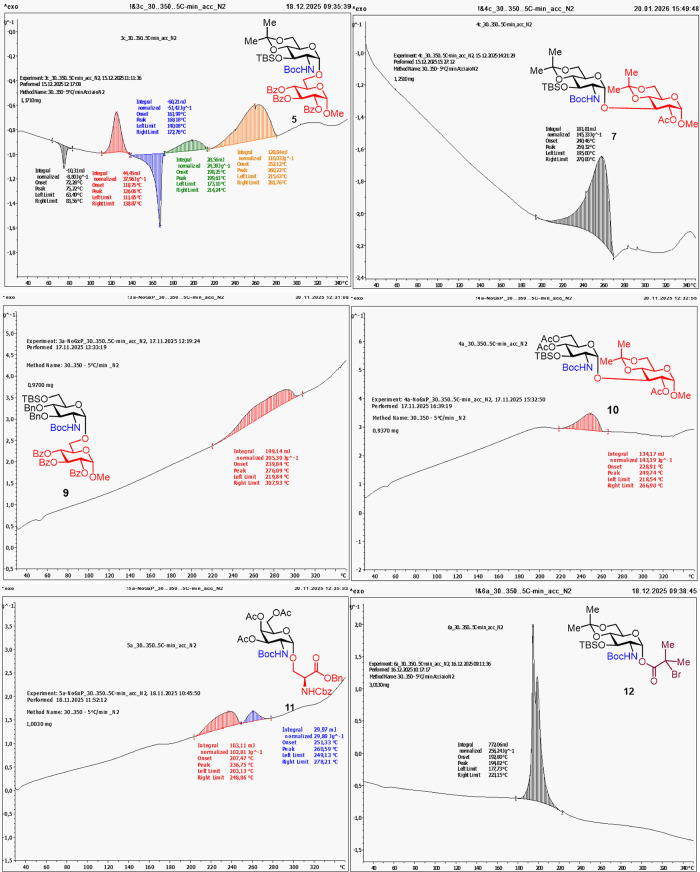
DSC analysis of the glycosylation products in the iron-catalyzed glycal 1,2-*cis*-aminoglycosylation.

We first evaluated the thermal stability of amination reagents **2a** and **2b** as they contain high-energy N–O bonds and are used in superstoichiometric amounts in the glycosylation. The DSC experiment concluded that amination reagent **2a** melts at 77 °C and starts to decompose at 122 °C (*T*_onset_: 154 °C, Δ*H*: −637 J/g) (Figure 5). A second exothermic event was observed starting around 188 °C (*T*_onset_: 190 °C, Δ*H*: −354 J/g), which is likely due to decomposition of the 2-bromoisobutyric acid moiety in **2a**. Amination reagent **2b**, derived from 2-acetoxyisobutyric acid, melts at 70 °C and shows only one exothermic event (*T*_onset_: 172 °C, Δ*H*: −610 J/g) in the thermogram (Figure 5). Notably, the decomposition onset temperatures of both **2a** and **2b** are significantly higher than the glycosylation temperature (−40 °C), providing a safety operating temperature margin (>100 °C) for large-scale operation.

The high heat release (>610 J/g) associated with the decomposition of **2a** and **2b** at high temperatures (>154 °C) suggested that autocatalytic processes might get involved during their decomposition.^50^ To make sure that **2a** and **2b** can be safely stored and used in bulk for iron-catalyzed glycosylation, we further evaluated their stability using ARC (Figure 6). Unlike *O*-benzoyl-*N*,*N*-dialkylhydroxylamines (BzO-NR_2_) **8a** and **8b**, studied by Valco,^44^ that spontaneously decompose upon phase transition and are thereby considered “melt-and-go” chemicals (Table 1),^44^ the onset decomposition temperatures of **2a**/**2b** are significantly higher than their melting points (77 and 70 °C, respectively). While any amorphous fraction in **8a** and **8b** could accelerate their decomposition upon melting, **2a** and **2b** are much more stable than BzO-NR_2_ in their neat state, at least 20 °C above their melting temperatures.

**Table 1:**
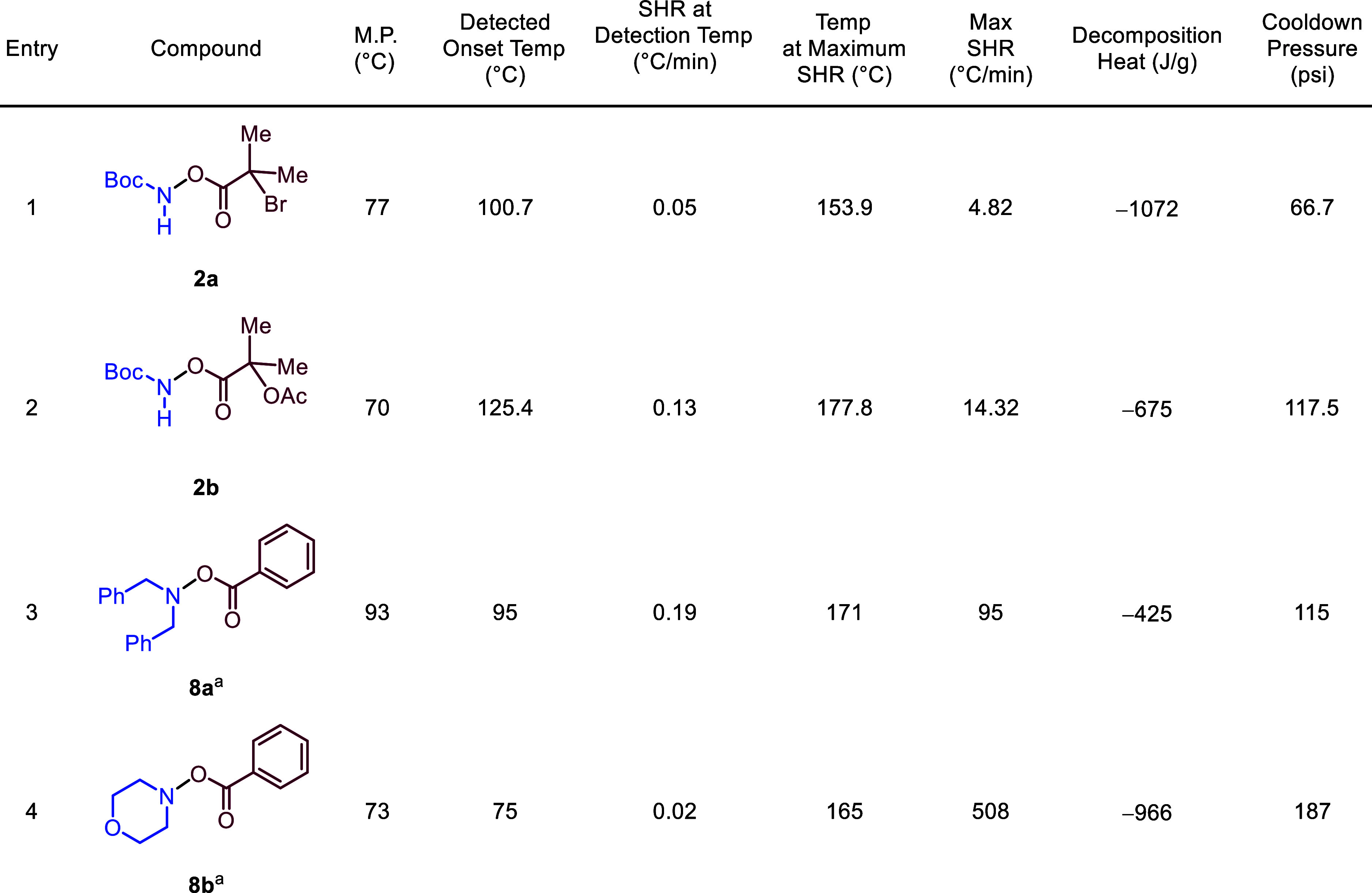
Comparison of ARC Test Results between **2a**/**2b** and BzO-NR_2_ **8a**/**8b**

a Data adapted from ref 44.

In ARC studies of **2a** and **2b**, we also noticed that the massive decomposition is preceded by a small thermal induction that has a decomposition rate higher than the global decomposition (Figure 6C,E). This rate difference suggested an autocatalytic reaction at the beginning of the decomposition, which is reminiscent of the ARC studies of BzO-NR_2_ (**8a**/**8b**) reported by Valco.^44^ However, distinct from **8a** and **8b** that present very high maximum self-heat rates (SHR) (Table 1, >95 °C/min), we observed modest maximum SHR for **2a** and **2b** (Table 1, 4.82 °C/min and 14.32 °C/min, respectively). These data are important for scaling up glycosylation involving the usage of **2a** and **2b** because they suggested moderately exothermic decomposition processes of **2a** and **2b** at high temperature (>100 °C). Notably, compared with BzO-NR_2_, **2a** and **2b** are less problematic for potential safety hazards from autocatalytic decomposition.

Moreover, pressure increase of the adiabatic system (gas generation) occurred simultaneously with initial decomposition of **2a** and **2b**, and its kinetics is very similar to the ones of their thermal decomposition (Figure 6D,F). However, for BzO-NR_2_ **8a**, gas evolution was not observed until 180 °C when a second maximum SHR of **8a** was observed.^44^

Our kinetic studies of this glycal *cis*-aminoglycosylation suggested that its initial rate has a first-order dependence on iron catalyst **1**, so we further evaluated the catalyst’s thermal stability (Figure 7). We observed that iron catalyst **1** is thermally stable, and there is no exothermic event up to 143 °C in an isochoric setting (*T*_onset_: 153 °C, Δ*H*: −293 J/g, Figure 7). Notably, two endothermic events were observed in an isobaric setting (*T*_onset_: 137 °C and Δ*H*: 196 J/g, *T*_onset_: 179 °C and Δ*H*: 128 J/g), presumably due to the decomposition of **1** with the development of gaseous products. Once again, the decomposition onset temperature of **1** is much higher (>100 °C) than the catalytic glycosylation temperature, which offers a comfortable temperature margin for safe large-scale operation.

Based upon these DSC results, we subsequently evaluated the mechanical impact sensitivities of iron catalyst **1** and amination reagents (**2a** and **2b**) by the Fall Hammer test (DWT). Gratifyingly, all of them uniformly demonstrate negative results in the DWT. These data confirm high stability of the product under any mechanical impact during the whole reaction process.

To ensure that the 1,2-*cis*-aminoglycosylation products are amenable to large-scale production, we also carried out the DSC analysis of two 1,2-*cis*-aminoglycosides (**5** and **7**) that were used in kinetic studies (Figure 8).

The DSC thermogram of aminoglycoside **5** displays a couple of mild endothermic and exothermic events up to 198 °C (Δ*H*: up to −24 J/g); however, a significant thermal decomposition was observed starting at 215 °C (*T*_onset_: 252 °C; Δ*H*: −110 J/g). Interestingly, aminoglycoside **7** displays only an exothermic event at a higher temperature in the DSC analysis (*T*_onset_: 240 °C; Δ*H*: −145 J/g). These results suggested that the 1,2-*cis*-aminoglycosylation products are thermally stable for handling at room temperature. Furthermore, we carried out DSC experiments for several other synthetically valuable aminoglycosides with distinct steric and electronic properties, including **9**, **10**, and **11**, and concluded that they are all thermally stable (Figure 8).

We further explored potential runaway scenarios in which one or more components were inadvertently omitted from iron-catalyzed glycosylation (Scheme 2). We observed that omission of a glycosyl acceptor resulted in formation of a 1,2-*cis*-glycal aminoacyloxylation product **12**.^39^ In the DSC analysis of **12**, only a single exothermic event was observed (*T*_onset_ = 190 °C, Δ*H*: −256 J/g, Figure 8). In the absence of a glycal donor, regardless of the presence of a glycosyl acceptor **4**, amination reagent **2a** was partially decomposed to BocNH_2_ (**13**) and 2-bromoisobutyric acid (**14**) with low conversion (<20% conversion). DSC analysis of **13** revealed two endothermic events (*T*_onset_ = 88 °C, Δ*H* = 22 J/g; *T*_onset_ = 228 °C, Δ*H* = 230 J/g), in addition to its melting at 108 °C (Figure S14). Collectively, these results suggested that the iron-catalyzed glycal 1,2-*cis*-aminoglycosylation does not exhibit hazardous exothermic behavior under plausible reagent-omission scenarios and that this glycosylation is amenable to large-scale operation.

**Scheme 2:**
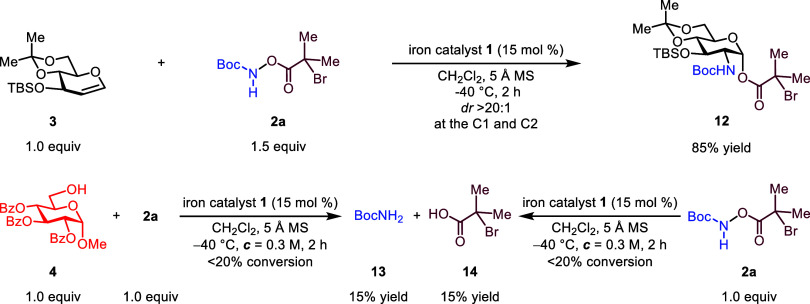
Controlled Runaway Reactions for the Iron-Catalyzed Glycal 1,2-*cis*-Aminoglycosylation

The observed thermal stability of 1,2-*cis*-amidogalactosyl serine benzyl ester **11** (Tn antigen) is particularly encouraging, as it is a key building block for *O*-linked glycopeptide synthesis. The synthesis of stereochemically pure Tn antigens is critically important for access to homogeneous *O*-linked glycopeptides. However, this has been challenging because the embedded 1,2-*cis*-amidoglycosidic linkage is difficult to form reliably in high stereoselectivity. The vast majority of existing syntheses of Tn antigens leverage non-neighboring participating groups, most notably the azido group, to enhance the *cis*-selectivity.^22,51–55^ However, structural variations either in galactosyl donors or amino acid acceptors often compromise the high 1,2-*cis*-selectivity, affording diastereomeric mixtures.^22,30,52–64^ Based upon this process safety assessment, we developed a 10 mmol scale, iron-catalyzed 1,2-*cis*-aminoglycosylation procedure for the synthesis of fully protected Tn antigen (**11**) (Scheme 3). The commercially available and bench-stable tri-*O*-acetyl-d-galactal (**15**) and *N*-Cbz serine benzyl ester (**16**) are readily glycosylated by iron catalyst **1** and amination reagent **2a**, affording protected Tn antigen **11** in high yield (6.09 g, 85% yield). Rapid and scalable postglycosylation deprotection procedures minimize aqueous workups and readily afford single diastereomeric Tn antigen (**17**).^40^

**Scheme 3:**
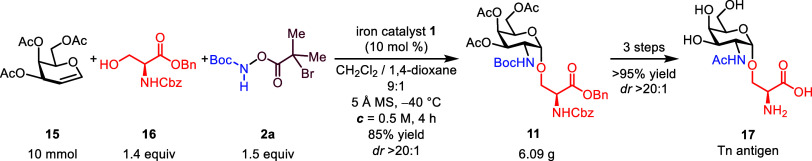
Stereoselective Multigram-Scale Tn Antigen Synthesis via the Iron-Catalyzed Glycal 1,2-*cis*-Aminoglycosylation

## Conclusions

In conclusion, we have carried out process safety assessment of the iron-catalyzed glycal 1,2-*cis*-aminoglycosylation process through DSC, ARC, and/or DWT of the amination reagents, iron catalysts, and representative glycosylation products. These experiments suggest that all of them are thermally stable at temperatures >100 °C above the glycosylation temperature and that the amination reagents are impact-stable and suitable for long-term storage in bulk. Although the decomposition enthalpies of these amination reagents are high at high temperatures (>100 °C), ARC analysis confirmed that their maximum SHR are modest. While the heat of the reaction is expected to be low under cryogenic conditions, calorimetry data will be obtained in future studies to ensure that any heat generated from the reaction can be controlled. Guided by these process safety studies, we have developed a multigram-scale, iron-catalyzed glycosylation process that readily affords stereochemically pure, biologically valuable Tn antigen.

## Experimental Section

### Differential Scanning Calorimetry

The DSC measurements were performed in a Mettler Toledo DSC1 using 40 μL aluminum punctured crucibles under a nitrogen atmosphere or 75 μL medium pressure PerkinElmer stainless steel crucibles under an air atmosphere. All measurements were carried out at a heating rate of 5 K/min.

To avoid the influence of internal pressure on degradation of the catalyst, DSC experiments were carried out utilizing a pierced pan.

### Accelerating Rate Calorimetry

The ARC experiments were carried out using a NETZSCH ARC 244 instrument. Approximately, 0.5 g of compound **2a** or 0.7 g of compound **2b** were placed in Thermal Hazard Technology Hastelloy C 0.025″ wall test-cell (Φ factors of the tests were of 6.9 and 5.0, respectively). The chosen Φ factors gave us the best results in terms of kinetic factors to compare the degradation pathways of compounds **2a** and **2b**. The samples were subjected to a step-shape heating profile, with a temperature increase of 5K for each step, until exotherm was detected (≥0.02 °C/min). Then, the instrument was switched into adiabatic mode following the temperature measured on the sample holder. Pressure changes were registered by a piezoelectric transducer within a typical operating maximum of 200 bar.

### Mechanical Sensitivity Test

The Fall Hammer Test (DWT) was designed to determine the sensitivity of potentially high explosive compounds, and it was carried out by dropping a 7 kg weight from the height of 0.5 m in accordance with the UN Recommendation on the Transport of Dangerous Goods, Manual of Tests and Criteria-Test 3 (a) (ii) as well as EN 13631-4.

### Procedure for the Multigram-Scale Iron-Catalyzed Glycal 1,2-*cis*-Aminoglycosylation of **16** with **15** and **2a**

To a flame-dried 100 mL round-bottom flask (flask **A**) equipped with a stir bar were added tri-*O*-acetyl-d-galactal (**15**) (2.72 g, 10 mmol, 1.0 equiv), *N*-Cbz serine benzyl ester (**16**) (4.61 g, 14 mmol, 1.4 equiv), iron catalyst Fe­(**L1**)­(BF_4_)_2_(MeCN)­(H_2_O)_2_ (**1**) (580 mg, 1 mmol, 10 mol %), and freshly activated 5 Å powdered molecular sieves (ca. 4 g). After the flask was evacuated and backfilled with N_2_ twice, anhydrous CH_2_Cl_2_ (8 mL) and freshly opened 1,4-dioxane (2 mL) were added, and the flask was cooled to −78 °C. To a flame-dried 25 mL round-bottom flask (flask **B**) was added acyloxyl carbamate **2a** (4.23 g, 15 mmol, 1.5 equiv). Flask **B** was evacuated and backfilled with N_2_ twice, and anhydrous CH_2_Cl_2_ (10 mL) was added. Then, the solution in flask **B** was transferred to flask **A** via a syringe for 10 min. The reaction was kept at −78 °C for an additional 3 min before being switched to −40 °C. The reaction was kept at −40 °C for 4 h and quenched by precipitating the iron catalyst with Et_2_O (40 mL) at the same temperature. The mixture was stirred for 2 min and subsequently warmed up to room temperature. The solution was then filtered through a short pad of Celite and washed with saturated aq NaHCO_3_ solution (15 mL). The organic phase was separated from the aqueous phase, which was further extracted with CH_2_Cl_2_ (15 mL × 3). The combined organic phase was dried over anhydrous Na_2_SO_4_ and concentrated in vacuo. The residue was purified through a silica gel flash column (hexanes/acetone: from 20:1 to 5:1) to afford the desired product **11** as a white foam (6.09 g, 85% yield).

## Supplementary Material



## Data Availability

All data generated or analyzed during this study are included in this published article and its Supporting Information file.

## Notes

Experimental procedure, characterization data for all new compounds, and selected NMR spectra (PDF)
