# Supplementary material for: Process Safety Assessment of the Iron-Catalyzed 1,2-cis-Selective Glycal Aminoglycosylation
Source: Org Process Res Dev. 2026 May 12;30(6):1642–51. doi: 10.1021/acs.oprd.6c00069 (PMC13186450; doi:10.1021/acs.oprd.6c00069)
Supplement: Supplementary file 1 [file op6c00069_si_001.pdf]

Supporting Information

Process Safety Assessment of the Iron-Catalyzed 1,2-*cis*-Selective Glycal Aminoglycosylation

Dakang Zhang,[‡a] Nicola Colombo,[‡b] Zixiang Jiang,[‡a] Davide Pirola,[‡b] Marino Nebuloni,*[b] and Hao Xu*[a]

[a]*Department of Chemistry, Brandeis University, 415 South Street, Waltham Massachusetts 02453, United States*

[b]*Redox Laboratory, Viale G.B. Stucchi 62/26, Monza (MB), 20900, Italy*

A. General Information

B. Introduction to the Stability Data

C. Results of the Stability Test

- **a. Materials and Methods**
- **b. Results and Discussion**

D. Multigram-Scale Synthesis of Fully Protected Tn Antigens via the Iron-Catalyzed Glycal 1,2-*cis*-Aminoglycosylation and Procedures for Post-glycosylation Transformations to Afford Tn Antigens and *O*-Galactosyl Amino Acids

E. References

F. NMR Spectra

A. General Information

General Procedures. All reactions were performed in oven-dried or flame-dried round-bottom flasks and vials. Stainless steel syringes and cannula were used to transfer air- and moisture-sensitive liquids. Flash chromatography was performed using silica gel 60 (230−400 mesh) from Sigma−Aldrich.

Materials. Commercial reagents were purchased from Sigma−Aldrich, TCI, Oakwood Chemicals, Combi-Blocks, Chem-Impex, Thermo Fischer Scientific and used as received. All solvents were used after being freshly distilled unless otherwise noted.

Instrumentation. Proton nuclear magnetic resonance (^1H NMR) spectra and carbon nuclear magnetic resonance (^{13}C NMR) spectra were recorded on Advance NEO 400 (400 MHz) and Varian 400-MR (400 MHz). Chemical shifts for protons are reported in parts per million downfield from tetramethylsilane and are referenced to the NMR solvent residual peak ($CHCl_3$ δ 7.26, CD_3OD δ 3.31). Chemical shifts for carbons are reported in parts per million downfield from tetramethylsilane and are referenced to the carbon resonances of the NMR solvent ($CDCl_3$ δ 77.0, CD_3OD δ 49.0). Data are represented as follows: chemical shift, multiplicity (br = broad, s = singlet, d = doublet, t = triplet, q = quartet, quint = quintet, m = multiplet), coupling constants in Hertz (Hz), and integration. The mass spectroscopic data were obtained at Brandeis Mass Spectrometry Facility using a Bruker timsTOF Pro instrument by electrospray ionization (ESI). Infrared (IR) spectra were obtained using a Nicolet IR200 spectrometer with a diamond ATR. Data are represented as follows: frequency of absorption (cm^{-1}) and absorption strength (s = strong, m = medium, w = weak). Optical rotations were measured on a Jasco P-2000 Polarimeter. The cuvette dimension is 10 cm and holds 1.5 mL.

B. Introduction to the Stability Data

A reactive chemical hazards assessment is the identification and quantification of dangerous energy release scenarios of a chemical process. This is typically accomplished by calculations and experimental testing.[1]

In many companies, a risk-consequence based approach is successfully applied to the testing strategy decision process. This approach balances the scale of operations (e.g., one-liter vessel in a R&D lab *vs*. a reactor in a plant) with the overall energy release potential.

A typical testing strategy is to screen first, and then apply. The term "screen" here means to apply less expensive, quicker turnaround, smaller scale, and less experimentally complicated tests. More sophisticated testing will be carried out if necessary.

Differential scanning calorimetry (DSC) is one of the most commonly applied thermal stability testing methodologies in reactive chemical hazards evaluation. In this study, the DSC was applied to assess the potential hazards of reagents, intermediates and final products in the iron-catalyzed olefin diazidation reaction.

It is also important to recognize that the overall energy release potential must contain two dimensions: thermodynamics (how much energy is released) and kinetics (how fast energy is released). The risk of the kinetics involves how "close" the process operates to a condition which would be hazardous. This concept has been explored in great details by Stoessel.[1f]

We also carried out the Drop Weight Test (DWT) on some of the compounds and assessed any potential hazard during the powder manipulation in the process.

C. Results of the Stability Test

a. Materials and Methods

Differential Scanning Calorimetry (DSC)

The DSC measurements were performed in a Mettler Toledo DSC1 using 40 µL aluminum punctured crucibles under nitrogen atmosphere or 75 µL medium pressure Perkin Elmer stainless steel crucibles under air atmosphere. All measurements were carried out at a heating rate of 5 K/min. To avoid the influence of internal pressure on degradation of catalyst, DSC experiments were also carried out utilizing pierced pan.

Accelerating Rate Calorimetry (ARC)

A Netzsch ARC 244 instrument was used for the ARC experiments. Approximately, 0.5 g of compound **2a** or 0.7 g of compound **2b** were placed in Thermal Hazard Technology Hastelloy C 0.025" wall test-cell, (Φ factors of the tests were of 6.9 and 5.0 respectively). The chosen Φ factors gave us the best results in term of kinetic factors to compare the degradation pathways of compounds **2a** and **2b**. The samples were subjected to a step-shape heating profile, with a temperature increase of 5K for each step, until exotherm was detected (≥0.02 °C/min), therefore instrument was switched into adiabatic mode following the temperature measured on the sample holder. Pressure changes were registered by a piezoelectric transducer within typical operating maximum of 200 bar.

Mechanical Sensitivity: Impact

The Fall Hammer Test (Drop Hammer) designed to determine the sensitivity of potentially high explosive compounds was carried out in accordance to the UN Recommendation on the Transport of Dangerous Goods, Manual of Tests and Criteria–Test 3 (a) (ii) as well as EN 13631–4.

The limiting impact energy is determined as the lowest energy at which a flash, flame, or explosion is observed. The test is used to assess the sensitivity of the test material to drop-weight impact.

The determination of the sensitivity to impact stimuli is one of the most important characteristics of energetic materials, which can be heat and shock-sensitive and can explosively decompose with little input of external energy. This determination is necessary to evaluate their safety in handling, processing or transportation.

In this manuscript, all tested substances were analyzed by dropping 7 Kg from 0.50 m height, i.e., 350 Kg×cm (35 Newton).

b. Results and Discussion

a) DSC Analysis of the Amination Reagents 2a and 2b

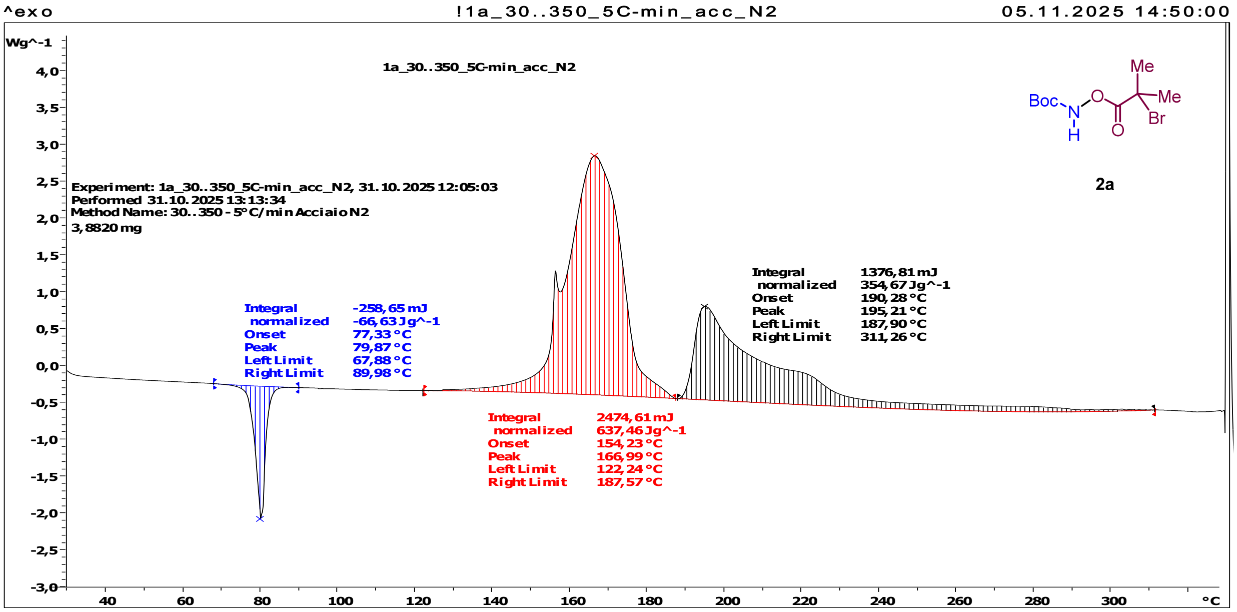

Figure S1. DSC Thermogram of Amination Reagent **2a** in Stainless-Steel Sealed Pan.

Amination reagent **2a** melts at 77 °C and starts to decompose at 122 °C (T_{onset}: 154 °C, ΔH: −637 J/g). A second exothermic event was observed starting around 188 °C (T_{onset}: 190 °C, ΔH: −354 J/g), which is likely due to decomposition of the 2-bromoisobutyric acid moiety in **2a** (Figure S1).

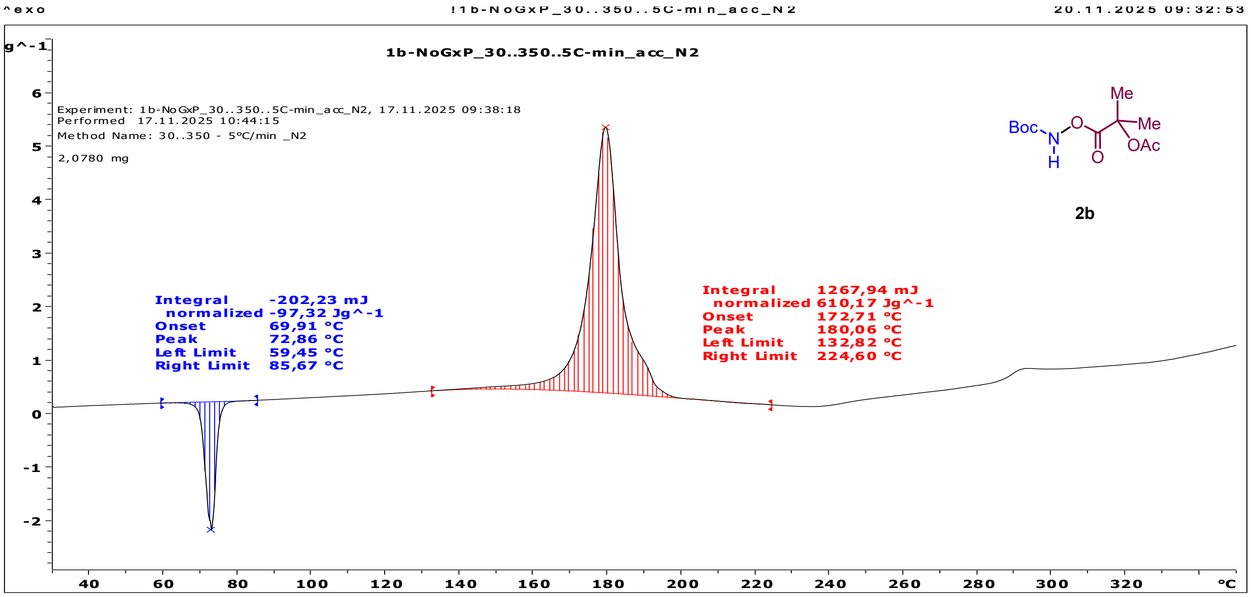

Figure S2. DSC Thermogram of Amination Reagent **2b** in Stainless-Steel Sealed Pan.

Amination reagent **2b** melts at 70 °C and shows only one exothermic event (T_{onset}: 172 °C, ΔH: −610 J/g) in the thermogram (Figure S2).

The decomposition onset temperatures of both **2a** and **2b** are significantly higher than the glycosylation temperature (−40 °C), providing a safety operating temperature margin (>100 °C) for large-scale operation.

b) ARC Analyses of the Amination Reagents 2a and 2b

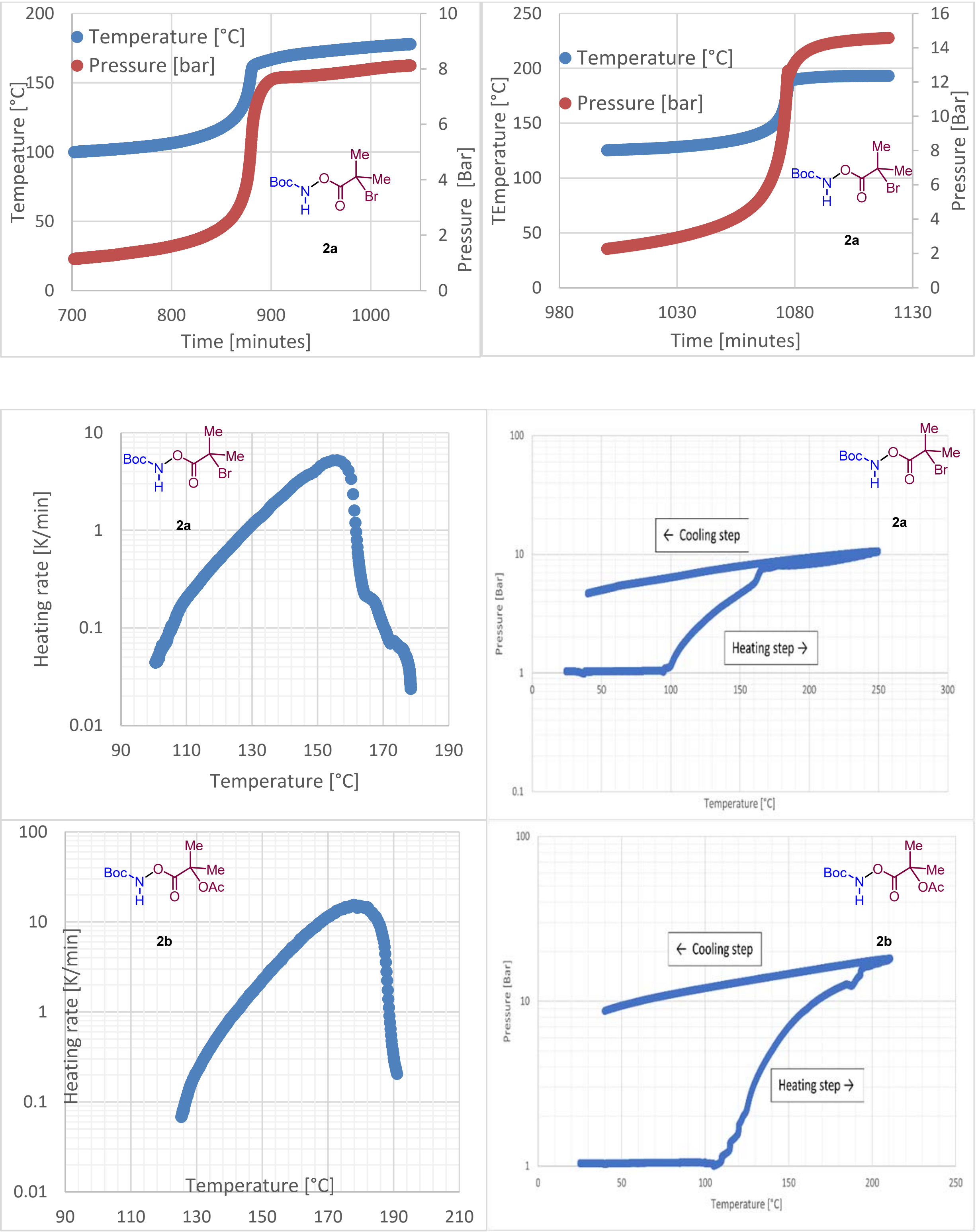

Figure S3. ARC Analysis of the Amination Reagents **2a**/**2b** in the Iron-Catalyzed Glycal 1,2-*cis*-Aminoglycosylation.

To make sure that **2a** and **2b** can be safely stored and used in bulk for the iron-catalyzed glycosylation, we further evaluated their stability using Accelerating Rate Calorimetry (ARC) (Figure S3). Unlike *O*-benzoyl-*N*,*N*-dialkylhydroxylamines (BzO–NR_2) **8a** and **8b** that spontaneously decompose upon phase transition and are thereby considered "melt-and-go" chemicals (Table S1),[2] the onset decomposition temperatures of **2a**/**2b** are significantly higher than their melting points (77 °C and 70 °C, respectively). These results suggested that both of them are more stable than BzO–NR_2 in their neat state above their melting temperatures.

In ARC studies of **2a** and **2b**, we also noticed that the massive decomposition is preceded by a small thermal induction that has a decomposition rate higher than the global decomposition (Figure S3). This rate difference suggested an autocatalytic reaction at the beginning of the decomposition, which is reminiscent of the recent ARC studies of BzO–NR_2 by Valco.[2] However, distinct from **8a** and **8b** that present very high maximum self-heat rates (SHR) (Table S1, >95 °C/min), we observed modest maximum SHR for **2a** and **2b** (Table S1, 4.82 °C/min and 14.32 °C/min, respectively), which suggested moderately exothermic decomposition processes of **2a** and **2b** and that both of them are less of safety concerns in terms of autocatalytic decomposition than BzO–NR_2.

It is also known that a second maximum SHR of **8a** was observed at 180 °C that accompanies a gas-generating process.[2] However, the ARC studies of **2a** and **2b** suggested that gas generation occurs simultaneously with their initial decomposition and that there is no observable second maximum SHR (Figure S3).

Table S1. Comparison of ARC Test Results Between **2a**/**2b** and **BzO–NR_2 8a**/**8b**

Entry	Compound	M.P. (°C)	Initial Temperature (°C)	SHR at Starting Temperature (°C/min)	Temperature at Maximum SHR (°C)	Max SHR (°C/min)	Decomposition Heat (J/g)	Cooldown Pressure (psi)
1	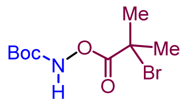 **2a**	77	100.7	0.05	153.9	4.82	–1072	66.7
2	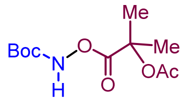 **2b**	70	125.4	0.13	177.8	14.32	–675	117.5
3	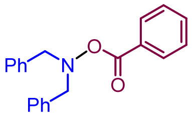 **Xa**[a]	93	95	0.19	171	95	–425	115
4	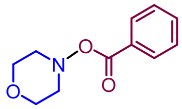 **Xb**[a]	73	75	0.02	165	508	–966	187

[a]Data adapted from reference 43.

c) ARC Analysis of the Amination Reagents 2a and 2b for Decomposition Kinetic Analysis

From a kinetic perspective, **2a** and **2b** exhibit essentially identical thermal behavior upon decomposition. Specifically, the initial portion of the heating-rate profiles (approximately the first 4–5 °C) shows a stepwise increase that differs from the subsequent region. This behavior suggests that the decomposition proceeds through a two-step mechanism.

In contrast to the DSC measurements, which revealed two distinct thermal events during the decomposition of compound **2a**, ARC analysis detected only a single event.

Furthermore, the kinetic parameters extracted from the ARC experiments and summarized in Table S2 are comparable for both compounds. This similarity supports the conclusion that compounds **2a** and **2b** undergo the same degradation pathway, consistent with their closely related molecular structures.

Table S2. Kinetic Parameter Evaluated by ARC for Compound **2a** and **2b**

Compounds	A [log(s^{-1})]	E_A [kJ/mol]	n
**2a**	25.882	225.682	1.416
**2b**	22.113	202.887	1.489

The kinetic parameters were calculated utilizing kinetic model included in the Netzsch Proteus Thermal Analysis software and disclosed by the following equation:

$$\frac{d\alpha}{dt} = A * (1-\alpha)^n * \exp(-\frac{E_A}{R * T})$$

Where A is the pre-exponential factor, α is the conversion degree, n is the order of reaction E_A is the activation energy of the reaction, R is the universal gas constant = 8.314 J/(mol *K) and T is the temperature.

d) DSC Analysis of the Amination Reagents 2d and 2e

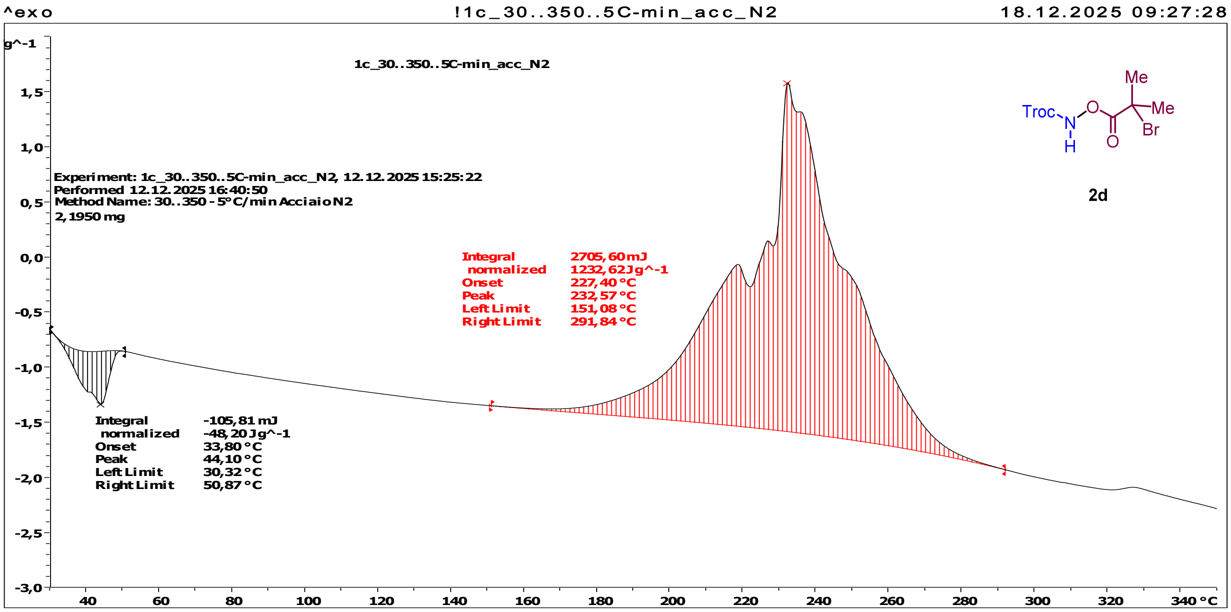

Figure S4. DSC Thermogram of Amination Reagent **2d** in Stainless-Steel Sealed Pan.

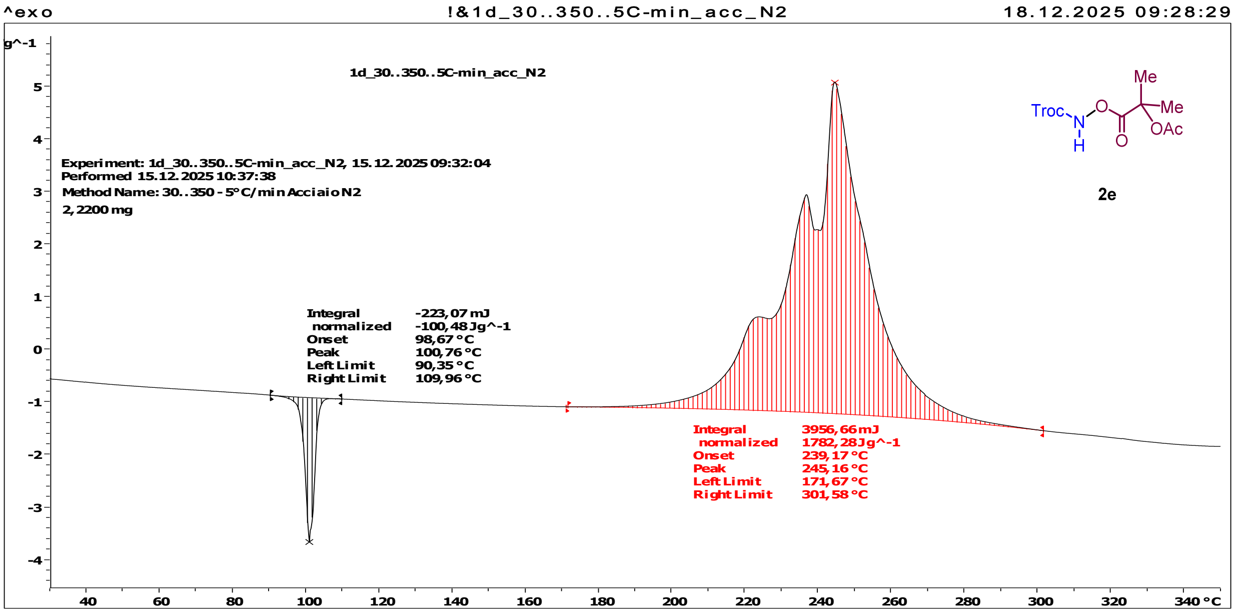

Figure S5. DSC Thermogram of Amination Reagent **2e** in Stainless-Steel Sealed Pan.

The *N*-Boc group is known to undergo spontaneous decarboxylation at elevated temperatures;[3] therefore, we next evaluated the thermal stability of the corresponding *N*-Troc–protected amination reagents **2d** and **2e** (Figures S4–S5). Notably, **2d** and **2e** are much less-efficient for the glycal 1,2-*cis*-aminoglycosylation which provides low conversions and low yields.[4] Surprisingly, both **2d** and **2e** exhibited markedly broader exothermic events in their DSC thermograms, spanning more than 130 °C, which were accompanied by substantially larger enthalpies of decomposition (ΔH: −1232 and −1782 J/g, respectively, Figure S4 and S5). These distinct thermal profiles suggested that the *N*-Boc-protected acyloxy carbamates (**2a** and **2b**) are thermally more stable than their *N*-Troc-protected analogs and that the presence of multiple halogen atoms may contribute to their decreased thermal stability.

e) DSC Analysis of Iron Catalyst 1

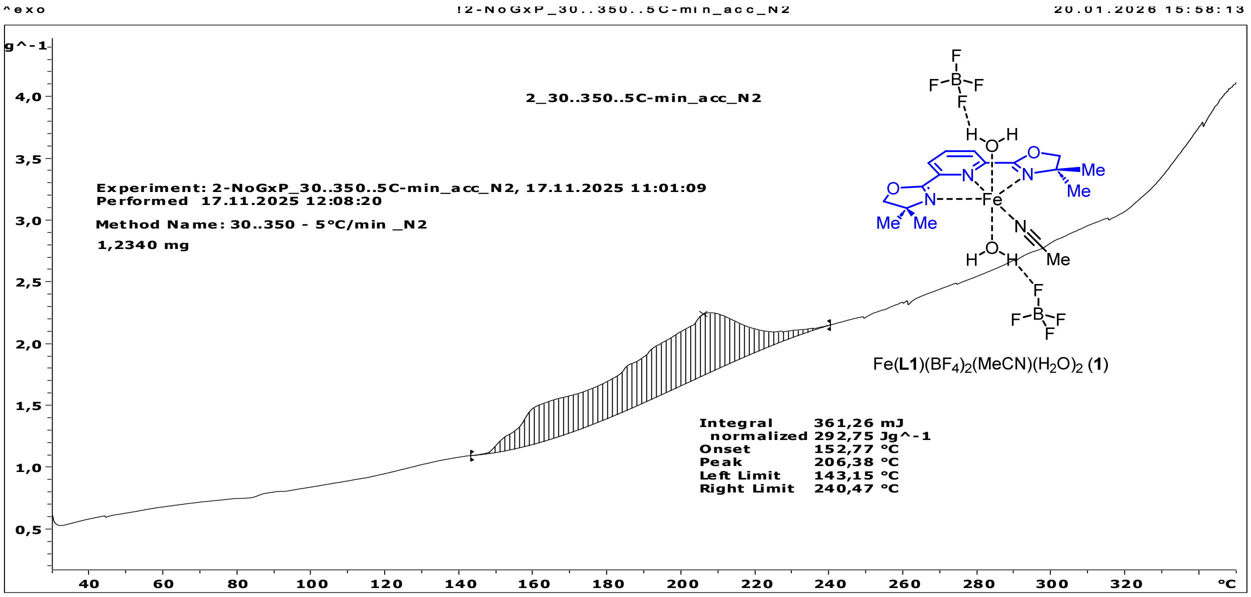

Figure S6. DSC Thermogram of Iron Catalyst **1** in Stainless-Steel Sealed Pan.

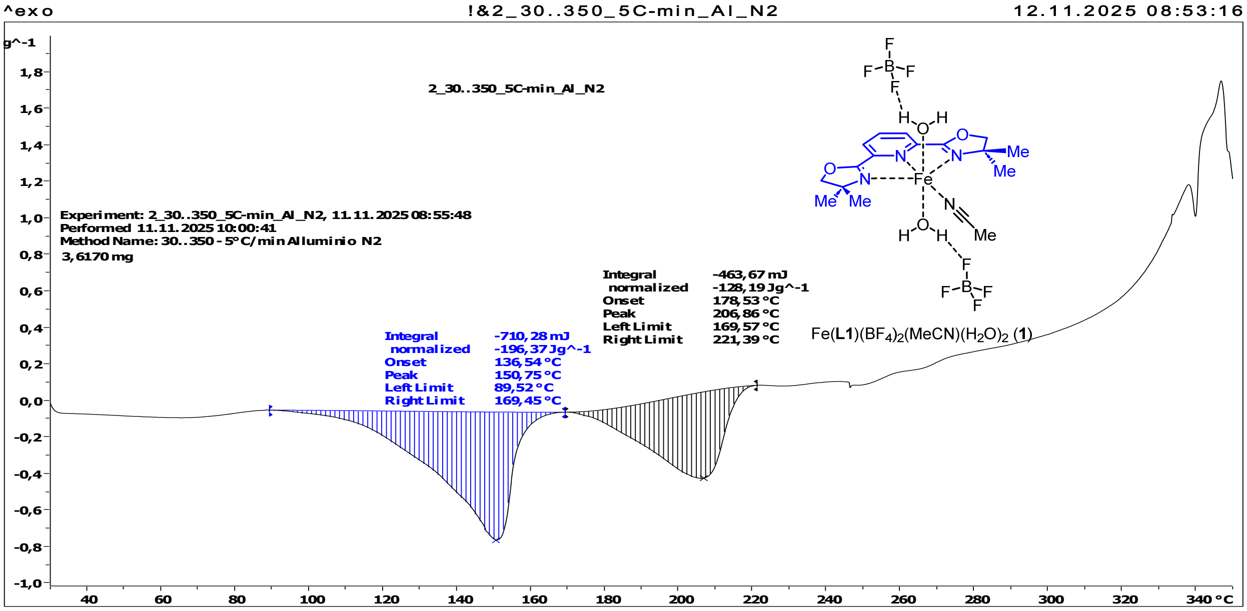

Figure S7. DSC Thermogram of Iron Catalyst **1** in Pierced Aluminum Pan.

Iron catalyst **1** is thermally stable and there is no exothermic event up to 143 °C in an isochoric setting (T_{onset}: 153 °C, ΔH: −293 J/g, Figure S6). Interestingly, two endothermic events were observed in an isobaric setting (T_{onset}: 137 °C and ΔH: 196 J/g, T_{onset}: 179 °C and ΔH: 128 J/g, Figure S7), presumably due to the decomposition of **1** with the development of gaseous products.

The decomposition onset temperature of **1** is much higher than the catalytic glycosylation temperature, which offers a comfortable temperature margin for safe large-scale operation.

f) Drop Weight Test of the Iron Catalyst and Amination Reagents

Based upon these DSC results, we subsequently evaluated the mechanic impact sensitivities of iron catalyst **1** and amination reagents (**2a** and **2b**) by the Fall Hammer Test (Drop Weight Test). Gratifyingly, all of them uniformly demonstrate negative results in the DWT. These data confirm higher stability of the product under any mechanical impact during the process product manipulation.

g) DSC Analysis of 1,2-*cis*-Aminoglycosides

The thermal stability of aminoglycosylation products (**5**, **7**, **9**, **10**, and **11**) was also assessed and the data were recorded and discussed as follows.

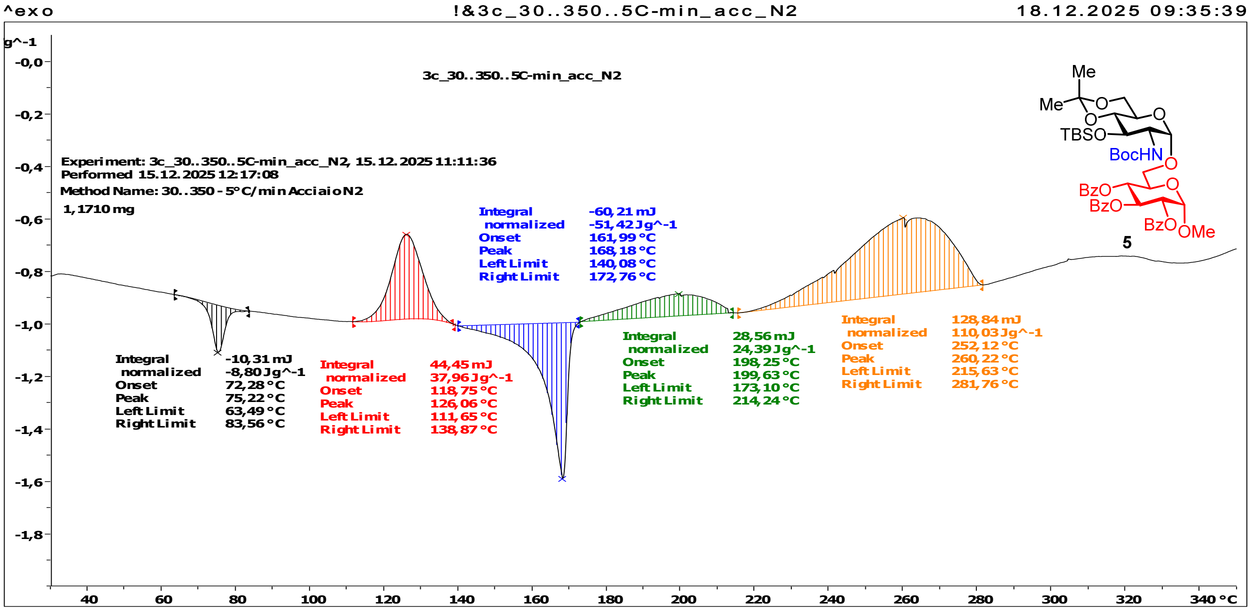

Figure S8. DSC Thermogram of Glycosylation Product **5** in Stainless-Steel Sealed Pan.

The DSC thermogram shows that the aminoglycoside **5** melts at 156 °C and there are a couple of mild endothermic and exothermic events up to 198 °C (ΔH: up to −24 J/g), presumably due to solid-solid transition (Figure S8). A significant thermal decomposition starting at 215 °C is also observed (T_{onset}: 252°C, ΔH: −110 J/g).

The thermal parameters for aminoglycoside **7**, **9**, **10**, and **11** are summarized in Table S3.

Table S3. DSC Parameters of Aminoglycoside **7**, **9**, **10**, and **11**

Aminoglycosides	Figure	Thermic Event.	Temperature		
		J / g	Range (°C)	Onset (°C)	Peak (°C)
**7**	S8	−145	195–270	240	260
**9**	S9	−205	220–308	240	276
**10**	S10	−147	219–267	229	250

**11**	S11	−103	203–249	207	237
		−30	249–278	251	261

Aminoglycoside **7**, **9**, **10**, and **11** are all thermally stable until 190 °C based on the DCS analysis. These results suggested that the 1,2-*cis*-aminoglycosylation products are safe for handling at room temperature.

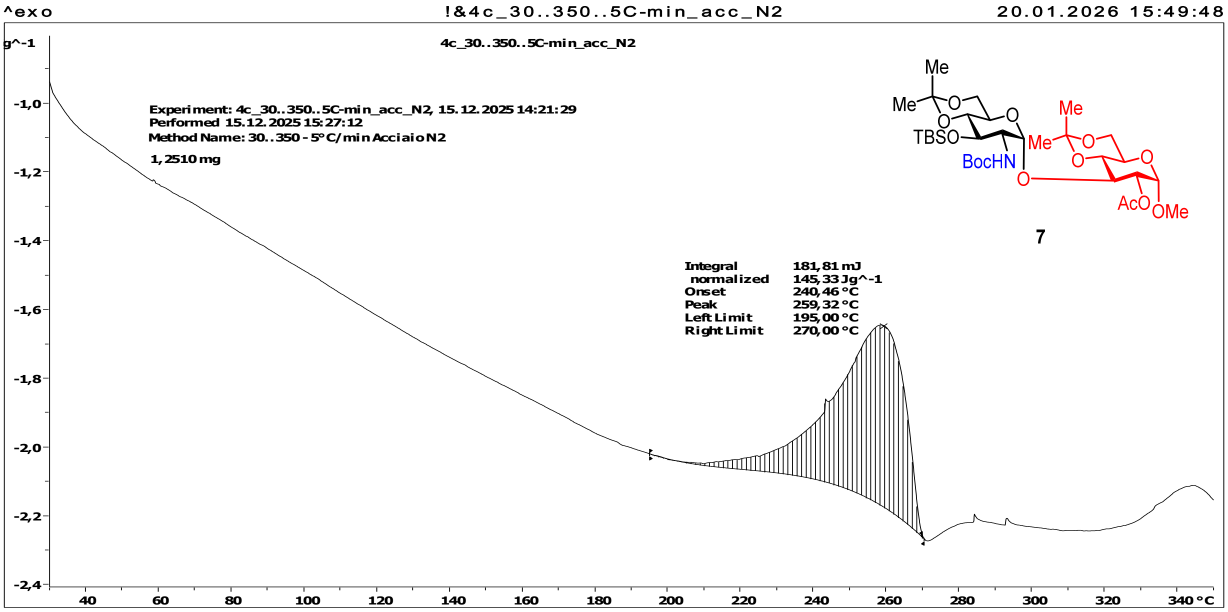

Figure S9. DSC Thermogram of Glycosylation Product **7** in Stainless-Steel Sealed Pan.

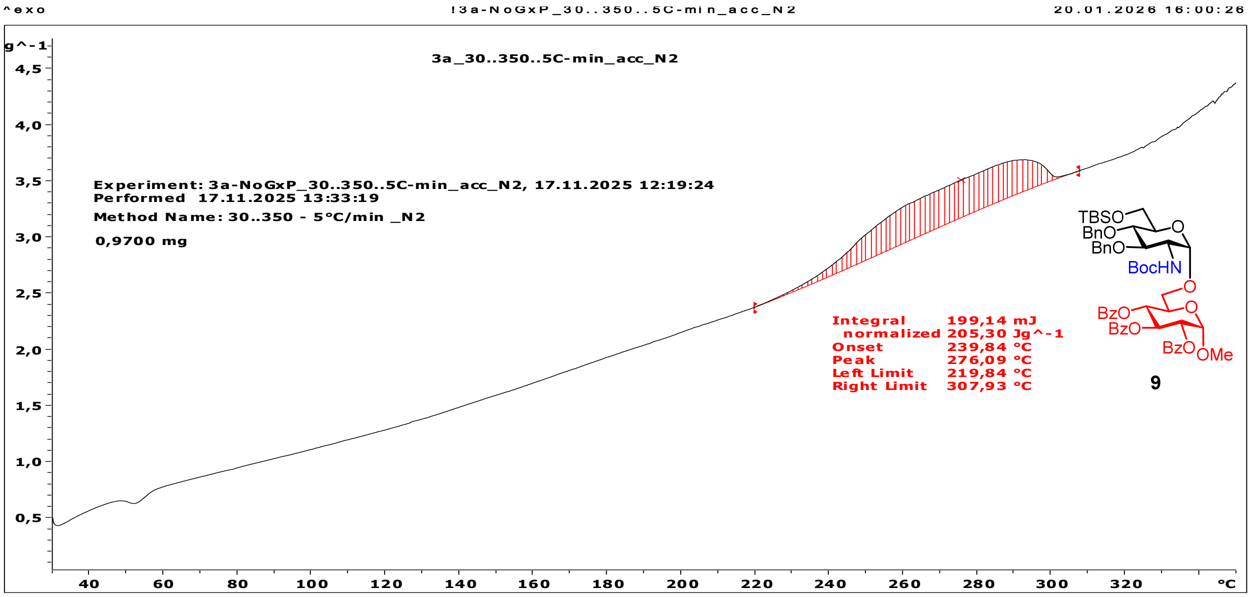

Figure S10. DSC Thermogram of Glycosylation Product **9** in Stainless-Steel Sealed Pan.

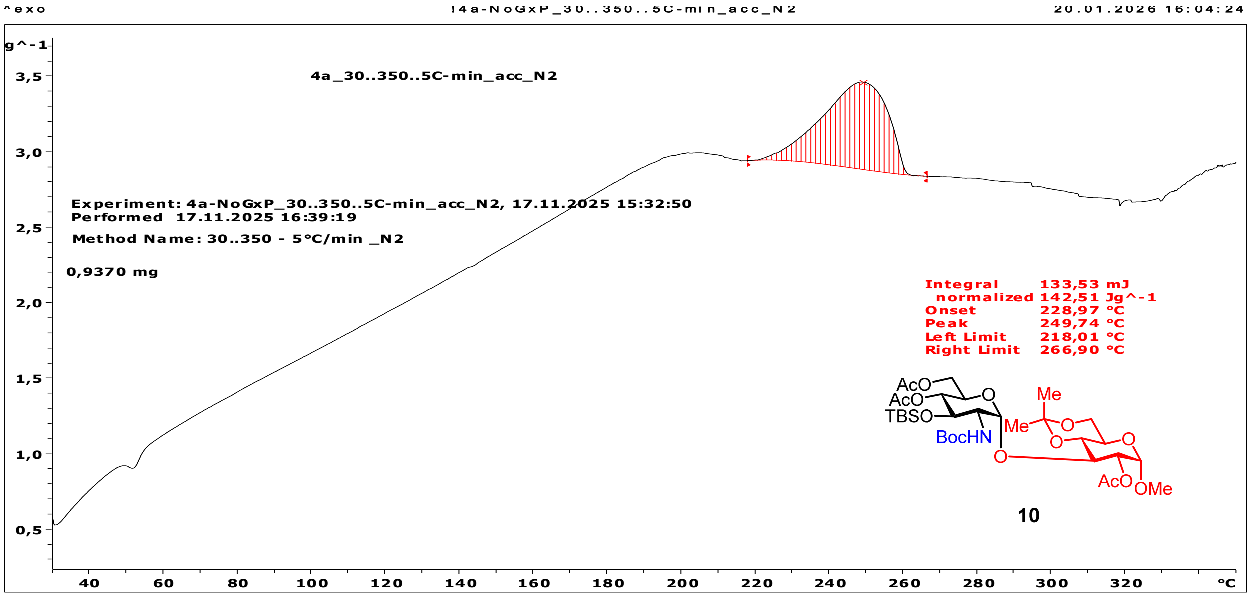

Figure S11. DSC Thermogram of Glycosylation Product **10** in Stainless-Steel Sealed Pan.

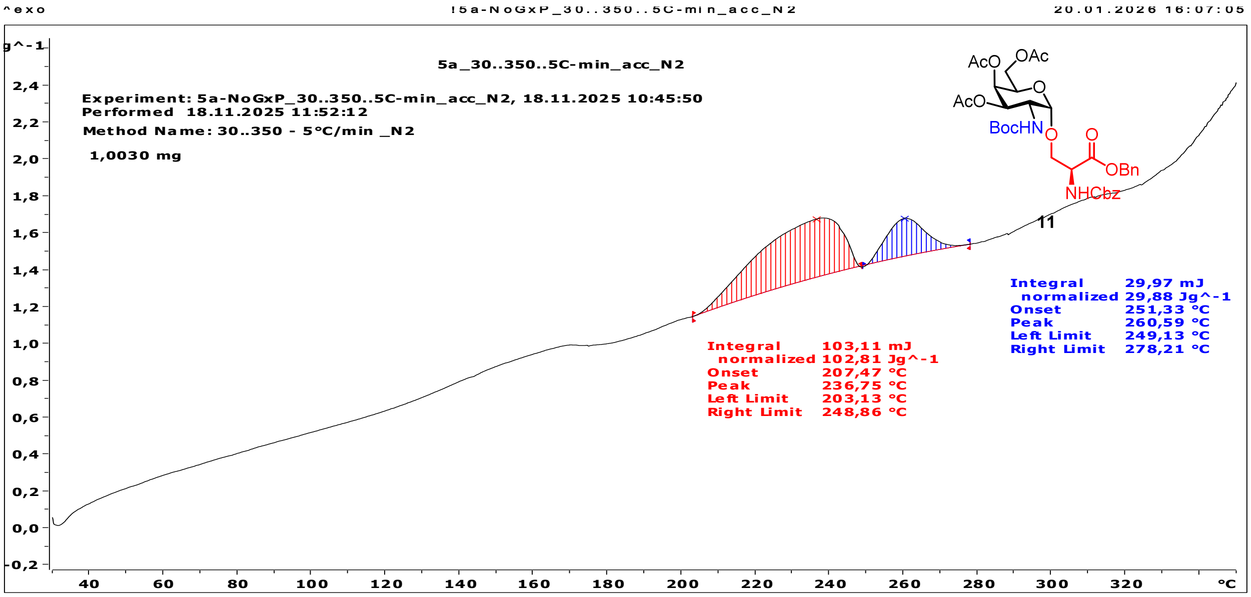

Figure S12. DSC Thermogram of Glycosylation Product **11** in Stainless-Steel Sealed Pan.

Lastly, the thermal stability of potential runaway reaction products (aminoacyloxylation product **12** and BocNH$_2$ (**13**) were also assessed and the data were recorded and discussed as follows.

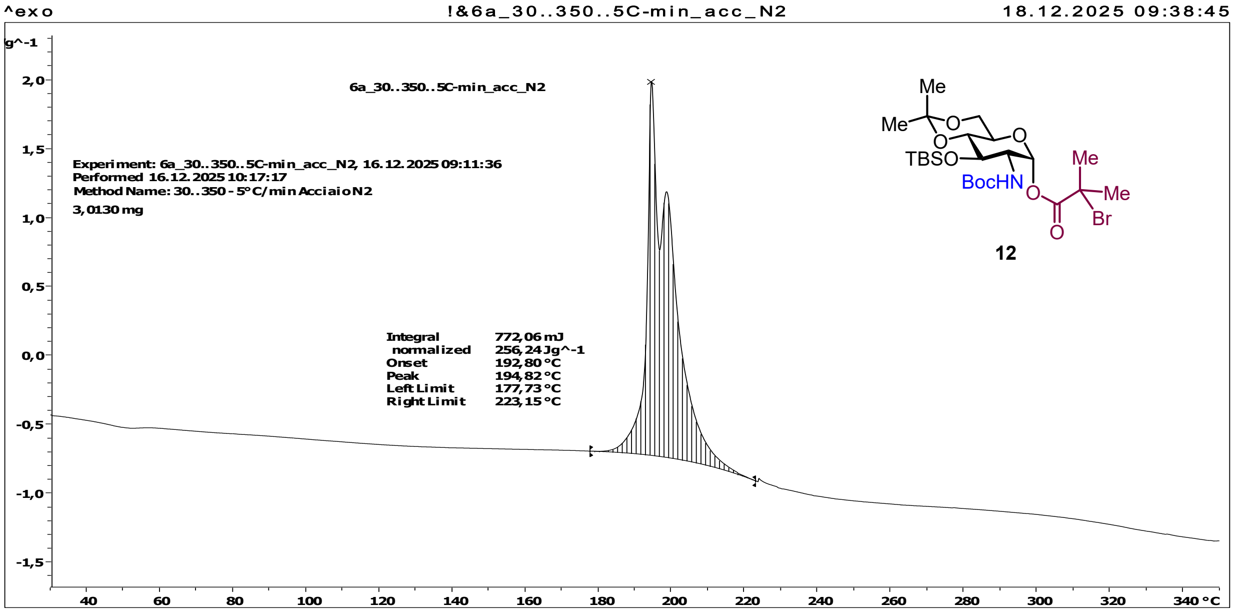

Figure S13. DSC Thermogram of 1,2-*cis*-Glycal Aminoacyloxylation Product **12** in Stainless-Steel Sealed Pan.

The thermogram display an exothermic event due to the decomposition of the product with T_{onset}: 193°C, T_{peak}: 195°C and ΔH: −256 J/g.

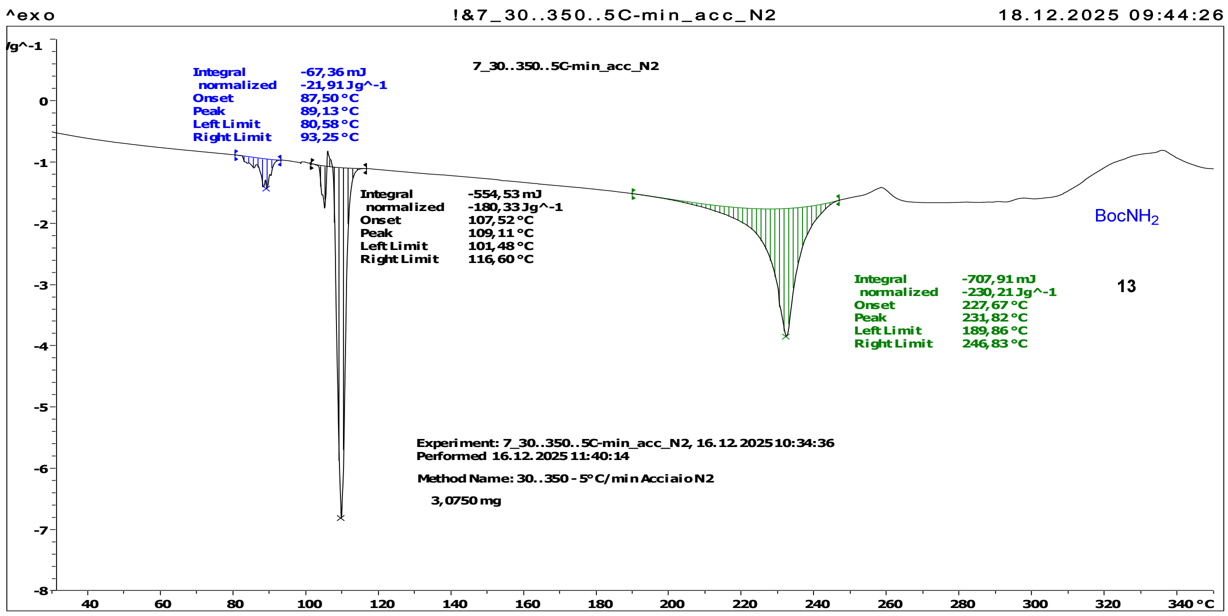

Figure S14. DSC Thermogram of $BocNH_2$ (**13**) in Stainless-Steel Sealed Pan.

DSC analysis of **13** revealed two endothermic events (T_{onset} = 88 °C, ΔH = 22 J/g; T_{onset} = 228 °C, ΔH = 230 J/g), in addition to its melting at 108 °C.

Collectively, these results suggested that the iron-catalyzed glycal 1,2-*cis*-aminoglycosylation does not exhibit hazardous exothermic behavior under plausible reagent-omission scenarios and that this glycosylation is amenable to large-scale operation.

D. Multigram-Scale Synthesis of Fully Protected Tn Antigens via the Iron-Catalyzed Glycal 1,2-cis-Aminoglycosylation and Procedures for Post-glycosylation Transformations to Afford Tn Antigens and O-Galactosyl Amino Acids

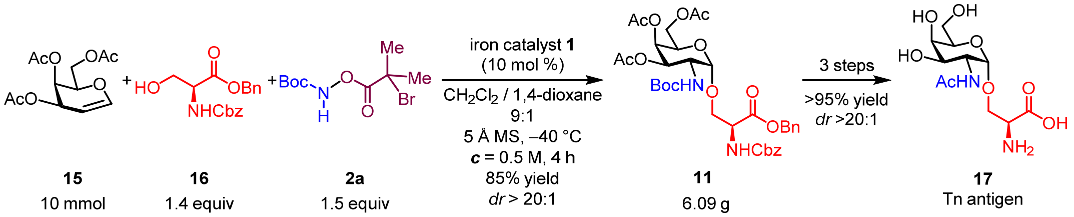

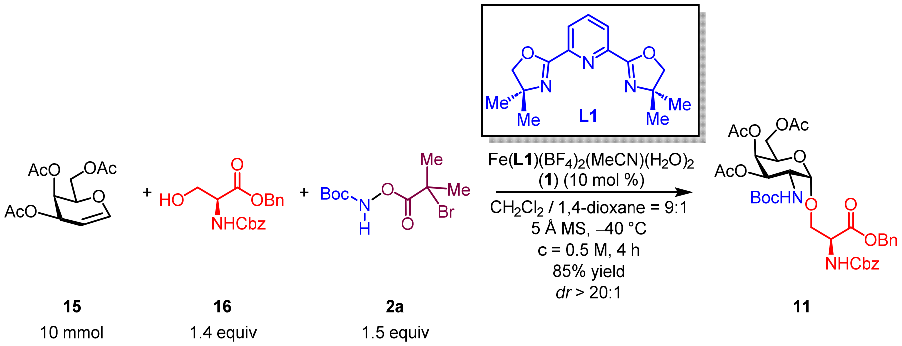

16 was synthesized according to a literature procedure.[5]

To a flame-dried 100 mL round bottom flask (flask **A**) equipped with a stir bar were added tri-*O*-acetyl-D-galactal (**15**) (2.72 g, 10 mmol, 1.0 equiv), *N*-Cbz serine benzyl ester (**16**) (4.61 g, 14 mmol, 1.4 equiv), iron catalyst Fe(**L1**)$(BF_4)_2(MeCN)(H_2O)_2$ (**1**) (580 mg, 1 mmol, 10 mol %), and freshly activated 5 Å powdered molecular sieves (*ca.* 4 g). After the flask was evacuated and backfilled with N_2 twice, anhydrous CH_2Cl_2 (8 mL) and freshly opened 1,4-dioxane (2 mL) were added and the flask was cooled to −78 °C. To a flame-dried 25 mL round bottom flask (flask **B**) was added acyloxyl carbamate **2a** (4.23 g, 15 mmol, 1.5 equiv). Flask **B** was evacuated and backfilled with N_2 twice and anhydrous CH_2Cl_2 (10 mL) was added. Then the solution in flask **B** was transferred to flask **A** via a syringe in 10 min. The reaction was kept at −78 °C for an additional 3 min before switched to −40 °C. The reaction was kept at −40 °C for 4 h and quenched by precipitating the iron catalyst with Et_2O (40 mL) at the same temperature. The mixture was stirred for two minutes and subsequently warmed up to room temperature. The

solution was then filtered through a short pad of Celite® and washed with saturated aq. $NaHCO_3$ solution (15 mL). The organic phase was separated from the aqueous one, which was further extracted with CH_2Cl_2 (15 mL × 3). The combined organic phase was dried over anhydrous Na_2SO_4 and concentrated *in vacuo*. The residue was purified through a silica gel flash column (hexanes/acetone: from 20:1 to 5:1) to afford the desired product **11** as white foam (6.09 g, 85% yield).[6]

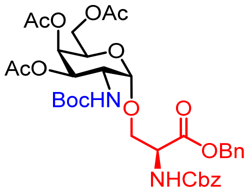

11

***N*-Benzyloxycarbonyl-*O*-(3,4,6-tri-*O*-acetyl-2-*tert*-butoxycarbonylamino-2-deoxy-α-D-galactopyranosyl)-L-serine benzyl ester (11)**: $[\alpha]_D^{22}$ +61.2 (acetone, *c* = 1.0); IR ν_{max} (neat)/cm^{-1}: 3357 (w), 2972 (w), 2360 (w), 1747 (s), 1716 (s), 1521 (m), 1456 (w), 1368 (m), 1226 (s), 1168 (m), 1133 (m), 1044 (m), 951 (w), 753 (w); ^{1}H NMR (400 MHz, $CDCl_3$) δ 7.42 – 7.30 (m, 10H), 5.80 (d, *J* = 8.3 Hz, 1H), 5.29 (d, *J* = 3.2 Hz, 1H), 5.25 – 5.19 (m, 2H), 5.12 (ABq, $\Delta\nu_{AB}$ = 13.5 Hz, J_{AB} = 12.2 Hz, 2H), 4.93 (dd, *J* = 11.3, 3.2 Hz, 1H), 4.79 (d, *J* = 3.8 Hz, 1H), 4.61 (d, *J* = 7.6 Hz, 1H), 4.54 (d, *J* = 10.2 Hz, 1H), 4.21 (td, *J* = 10.7, 3.7 Hz, 1H), 4.06 (d, *J* = 5.2 Hz, 1H), 4.03 – 3.92 (m, 4H), 2.14 (s, 3H), 2.00 (s, 3H), 1.99 (s, 3H), 1.41 (s, 9H); ^{13}C NMR (100 MHz, $CDCl_3$) δ 170.4 (two peaks overlapped, 2C), 170.2, 169.7, 155.8, 155.2, 136.0, 134.8, 128.8 (two peaks overlapped, 4C), 128.6 (2C), 128.5 (2C), 128.3, 128.1, 99.7, 80.0, 70.3, 68.8, 67.8, 67.3 (two peaks overlapped, 2C), 67.2, 61.9, 54.5, 48.8, 28.2 (3C), 20.7, 20.64, 20.60; HRMS: m/z (ESI) calcd for $C_{35}H_{45}N_2O_{14}^+$, $[M + H]^+$, 717.2865, found 717.2872. $^1J_{^{13}C1-H1}$ = 170.8 Hz.

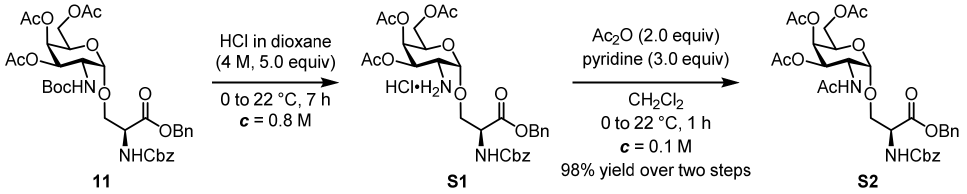

To a 100 mL oven-dried round-bottom flask equipped with a magnetic stirring bar was added compound **11** (4 g, 5.58 mmol, 1.0 equiv). The vial was cooled to 0 °C, followed by addition of HCl (4 M solution in 1,4-dioxane, 6.98 mL, 27.90 mmol, 5.0 equiv). The reaction mixture was stirred for 10 min at 0 °C before being warmed to room temperature. The reaction mixture was stirred for another 7 h with the progress monitored by TLC until completion. The reaction mixture was concentrated *in vacuo* with a base tube to afford the desired product **S1** as white foam which was directly used in the next step without further purification.

To the same flask containing the crude product (5.58 mmol, 1.0 equiv) from the previous step were added anhydrous CH_2Cl_2 (56 mL) and pyridine (1.35 mL, 16.74 mmol, 3.0 equiv). After the mixture was stirred for 3 min at 0 °C, Ac_2O (1.06 mL, 11.16 mmol, 2.0 equiv) was added dropwise. The reaction mixture was slowly warmed to room temperature and stirred for 1 h with the progress monitored by TLC until completion. The reaction mixture was then quenched with saturated aqueous NH_4Cl solution (32 mL). The organic phase was separated from the aqueous one, which was further extracted with CH_2Cl_2 (40 mL × 3). The combined organic layer was washed with brine, dried over anhydrous Na_2SO_4, and concentrated *in vacuo.* The desired product **S2** was obtained through column chromatography (hexanes/acetone: from 20:1 to 3:2) as white foam (3.6 g, 98% yield over two steps).[6]

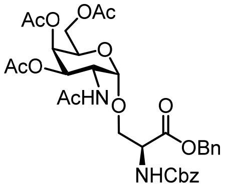

S2

***N*-Benzyloxycarbonyl-*O*-(2-acetamido-3,4,6-tri-*O*-acetyl-2-deoxy-α-D-galactopyranosyl)-L-serine benzyl ester** (**S2**): $[\alpha]_D^{22}$ +77.2 (acetone, *c* = 1.0); IR ν_{max} (neat)/cm^{-1}: 3355 (w), 2970 (w), 2360 (w), 1746 (s), 1522 (w), 1372 (w), 1230 (s), 1050 (m); ^1H NMR (400 MHz, $CDCl_3$) δ 7.34 (m, 10H), 5.82 (d, *J* = 8.3 Hz, 1H), 5.60 (d, *J* = 9.5 Hz, 1H), 5.30 (d, *J* = 3.2 Hz, 1H), 5.18 (ABq, $\Delta\nu_{AB}$ = 12.0 Hz, J_{AB} = 11.9 Hz, 2H), 5.12 (s, 2H), 5.02 (dd, *J* = 11.4, 3.2 Hz, 1H), 4.78 (d, *J* = 3.7 Hz, 1H), 4.60 (d, *J* = 8.3 Hz, 1H), 4.51 (ddd, *J* = 11.3, 9.5, 3.7 Hz, 1H), 4.04 (m, 3H), 3.97 –

3.86 (m, 2H), 2.14 (s, 3H), 1.99 (s, 3H), 1.98 (s, 3H), 1.89 (s, 3H); ^{13}C NMR (100 MHz, $CDCl_3$) δ 170.8, 170.4, 170.2, 170.1, 169.9, 155.8, 135.9, 134.7, 128.82 (2C), 128.80 (2C), 128.6 (2C), 128.4 (2C), 128.3, 128.2, 99.0, 69.7, 68.2, 67.7, 67.3, 67.2, 67.1, 61.9, 54.5, 47.6, 23.1, 20.70, 20.67, 20.6; HRMS: m/z (ESI) calcd for $C_{32}H_{39}N_2O_{13}^+$, $[M + H]^+$, 659.2447, found 659.2429. $^1J_{^{13}C1\text{–}H1}$ = 175.5 Hz.

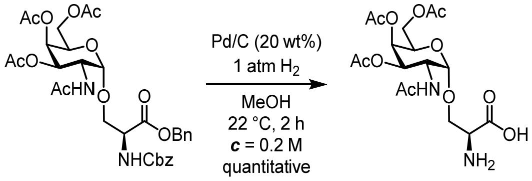

S2 **S3**

To a 100 mL round-bottom flask equipped with a magnetic stirring bar was added compound **S2** (3.6 g, 5.46 mmol, 1.0 equiv). After the flask was evacuated and backfilled with N_2 three times, Pd/C (720 mg, 20 wt %) was added. The flask was once again evacuated and backfilled with N_2 three times before MeOH (27.4 mL) was added. The flask was then evacuated and backfilled with H_2 three times and a H_2 atmosphere (1 atm) was maintained with a hydrogen balloon. The solution was stirred for 2 h at room temperature, with progress monitored by TLC until completion. The reaction mixture was then filtered through a short pad of Celite® (washed with MeOH), and the filtrate was concentrated *in vacuo* to afford the desired product **S3** (2.38 g, quantitative yield) as a white solid.[6]

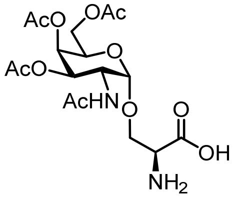

S3

***O*-(2-Acetamido-3,4,6-tri-*O*-acetyl-2-deoxy-α-D-galactopyranosyl)-L-serine** (**S3**): $[\alpha]_D^{22}$ +89.9 (methanol, *c* = 1.0); IR ν_{max} (neat)/cm^{-1}: 2970 (w), 2583 (w), 2360 (s), 2342 (m), 1792 (s), 1559 (s), 1374 (m), 1229 (s), 1053 (m); ^{1}H NMR (400 MHz, CD_3OD) δ 5.42 (d, *J* = 2.5 Hz, 1H), 5.20

(dd, J = 11.5, 3.2 Hz, 1H), 4.93 (d, J = 3.6 Hz, 1H), 4.50 (dd, J = 11.5, 3.6 Hz, 1H), 4.38 – 4.25 (m, 1H), 4.18 (dd, J = 11.1, 6.5 Hz, 1H), 4.15 – 4.04 (m, 2H), 3.92 – 3.82 (m, 2H), 2.15 (s, 3H), 2.04 (s, 3H), 1.98 (s, 3H), 1.95 (s, 3H); ^{13}C NMR (100 MHz, CD_3OD) δ 173.6, 172.2, 172.1, 172.0, 171.2, 100.1, 69.7, 68.6, 68.4, 68.3, 62.8, 55.9, 48.7, 22.8, 20.63, 20.59, 20.5; HRMS: m/z (ESI) calcd for $C_{17}H_{27}N_2O_{11}^+$, [M + H]$^+$, 435.1609, found 435.1601. $^1J^{13}{}_{C1–H1}$ = 172.2 Hz.

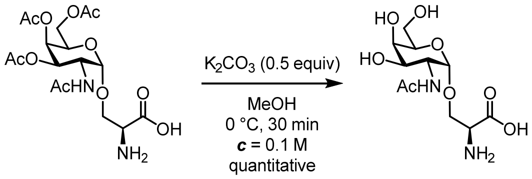

S3 **17**

To a 250 mL round-bottom flask equipped with a magnetic stirring bar were added compound **S3** (2.38 g, 5.46 mmol, 1.0 equiv) and MeOH (54.6 mL). The solution was cooled to 0 °C, and K_2CO_3 (powder, 378 mg, 2.73 mmol, 0.5 equiv) was then added. The reaction mixture was stirred for 30 min at 0 °C, with progress monitored by TLC until completion. The reaction mixture was then diluted with MeOH (40 mL) and neutralized with Amberlite® IRC 120 H. The reaction mixture was then filtered through a piece of cotton (washed with MeOH), and the filtrate was concentrated *in vacuo* to afford the desired product **17** (1.68 g, quantitative yield) as a white solid (m.p. 199–201 °C).[6]

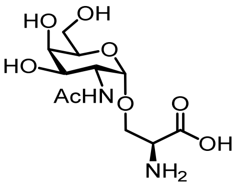

17

***O*-(2-Acetamido-2-deoxy-α-D-galactopyranosyl)-L-serine** (**17**): $[\alpha]_D^{22}$ +194.8 (methanol, c = 0.36); IR ν_{max} (neat)/cm^{-1}: 3283 (m), 2926 (m), 2569 (w), 2360 (s), 2342 (m), 1734 (m), 1700 (s), 1617 (m), 1374 (m), 1229 (m), 1053 (m); ^{1}H NMR (400 MHz, CD_3OD) δ 4.82 (d, J = 3.7 Hz, 1H), 4.32 (dd, J = 11.0, 3.7 Hz, 1H), 4.07 (dd, J = 10.9, 3.1 Hz, 1H), 3.92 – 3.81 (m, 3H), 3.78 (m, 3H), 3.70 (dd, J = 11.3, 4.6 Hz, 1H), 2.02 (s, 3H); ^{13}C NMR (100 MHz, CD_3OD) δ 174.1,

172.1, 100.2, 73.1, 70.4, 69.7, 68.6, 63.0, 56.2, 51.2, 22.9; HRMS: m/z (ESI) calcd for $C_{11}H_{21}N_2O_8^+$, [M + H]$^+$, 309.1292, found 309.1289. $^1J^{13}_{C1–H1}$ = 170.7 Hz.

E. References

1. (a) Crowl, D. A.; Louvar, J. F. *Chemical Process Safety*: *Fundamentals with Applications*. Prentice Hall, 1990; (b) Yoshida, T. *Safety of Reactive Systems*, Elsevier, 1987; (c) Grewer, T. *Thermal hazards of Chemical Reactions*, Elsevier, 1994; (d) Frurip, D. J.; Britton, L.; Fenlon, W.; Going, J.; Harrison, B. K.; Niemeier, J.; Ural, E. The Role of ASTM E27 Methods in Hazard Assessment. In *Proceedings of the 38th AIChE Loss Prevention Symposium*, New Orleans LA, 2004; (e) Frurip, D. J.; Elwell, T. Effective use of Differential Scanning Calorimetry in Reactive Chemicals Hazard Evaluation, Process Safety Prog.,AIChE, **2007**, *26*, 51; (f) Stoessel, F.; Ubrich, O. *J. Thermal Analysis and Calorimetry*, **2001**, 1, *64*; (g) Urben, P.; Bretherick L. *Bretherick's Handbook of Reactive Chemical Hazards*, Elsevier: Butterworth-Heinemann, 1999.
2. Topczewski, J. J.; Cheek, H.; Gruber, J. M.; Marsh, B. M.; Valco, D. J., Thermal Hazard Evaluation and Safety Considerations for the Use of *O*-Benzoyl-*N*-alkyl Hydroxylamines as Synthetic Reagents. *Org. Process Res. Dev.* **2025**, *29*, 2497-2503.
3. Ryan, M.-R.; Lynch, D.; Collins, S. G.; Maguire, A. R., Selective Thermal Deprotection of *N*-Boc Protected Amines in Continuous Flow. *Org. Process Res. Dev.* **2024**, *28*, 1946-1963.
4. Li, H.; Zhang, D.; Li, C.; Yin, L.; Jiang, Z.; Luo, Y.; Xu, H., Stereoselective Glycosylation for 1,2-*cis*-Aminoglycoside Assembly by Cooperative Atom Transfer Catalysis. *J. Am. Chem. Soc.* **2024**, *146*, 33316–33323.
5. Khaled, A.; Gravier-Pelletier, C.; Le Merrer, Y., Synthesis of Bis-(2,3,4,6-Tetra-*O*-Acetyl-α-D-Mannopyranosyl)-L-Serinyl Phosphate, as a Prodrug of Mannose-1-Phosphate. *Tetrahedron: Asymmetry* **2007**, *18*, 2121-2124.
6. Yin, L.; Zhang, D.; Jiang, Z.; Xu, H., Stereoselective Multigram-Scale Tn Antigen Synthesis via the Iron-Catalyzed Glycal 1,2-*cis*-Aminoglycosylation. *Org. Lett.* **2025**, *27*, 5515–5520.

F. NMR Spectra

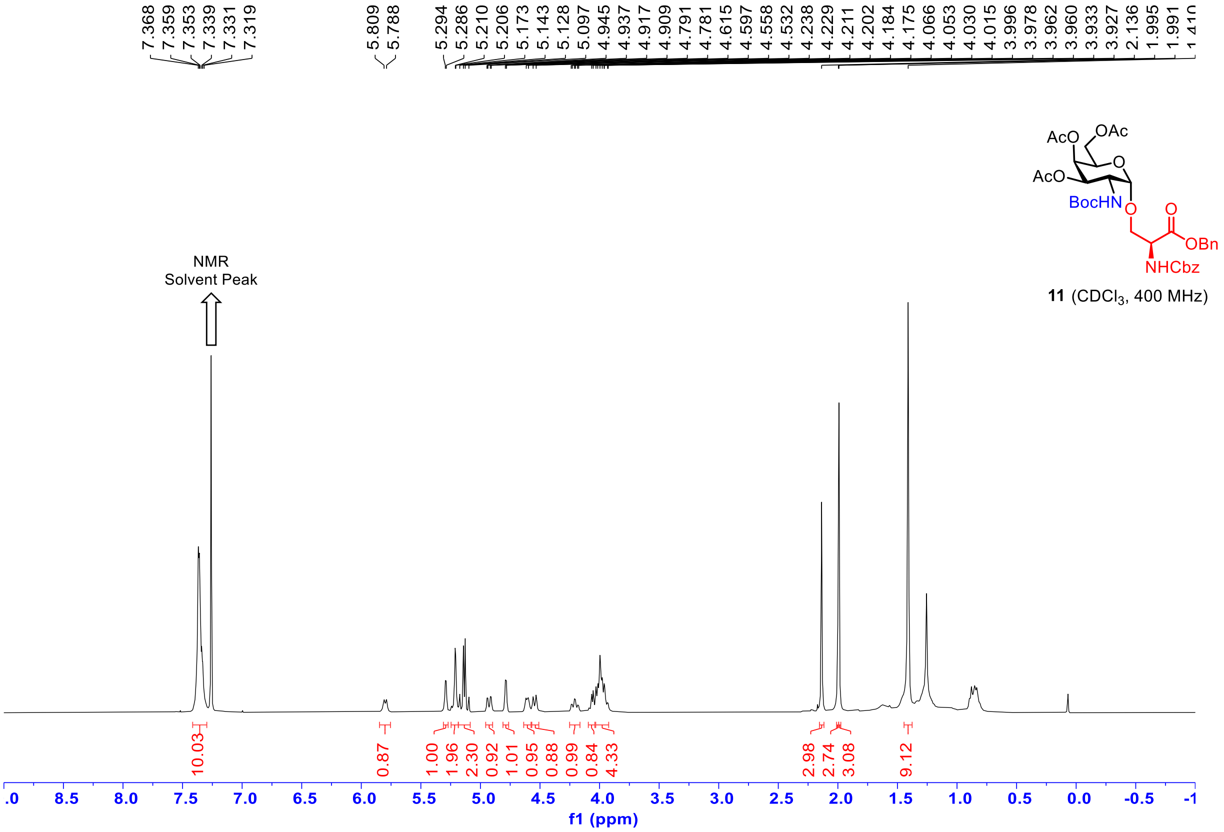

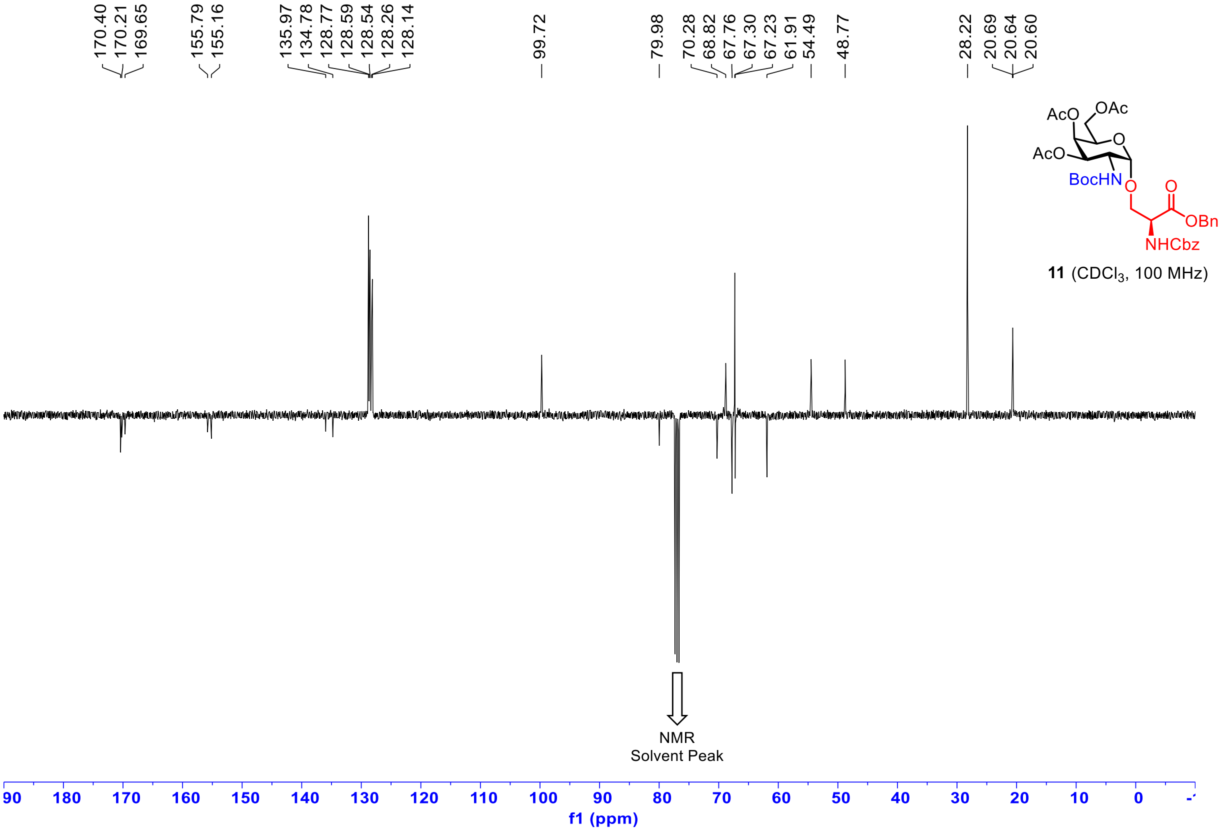

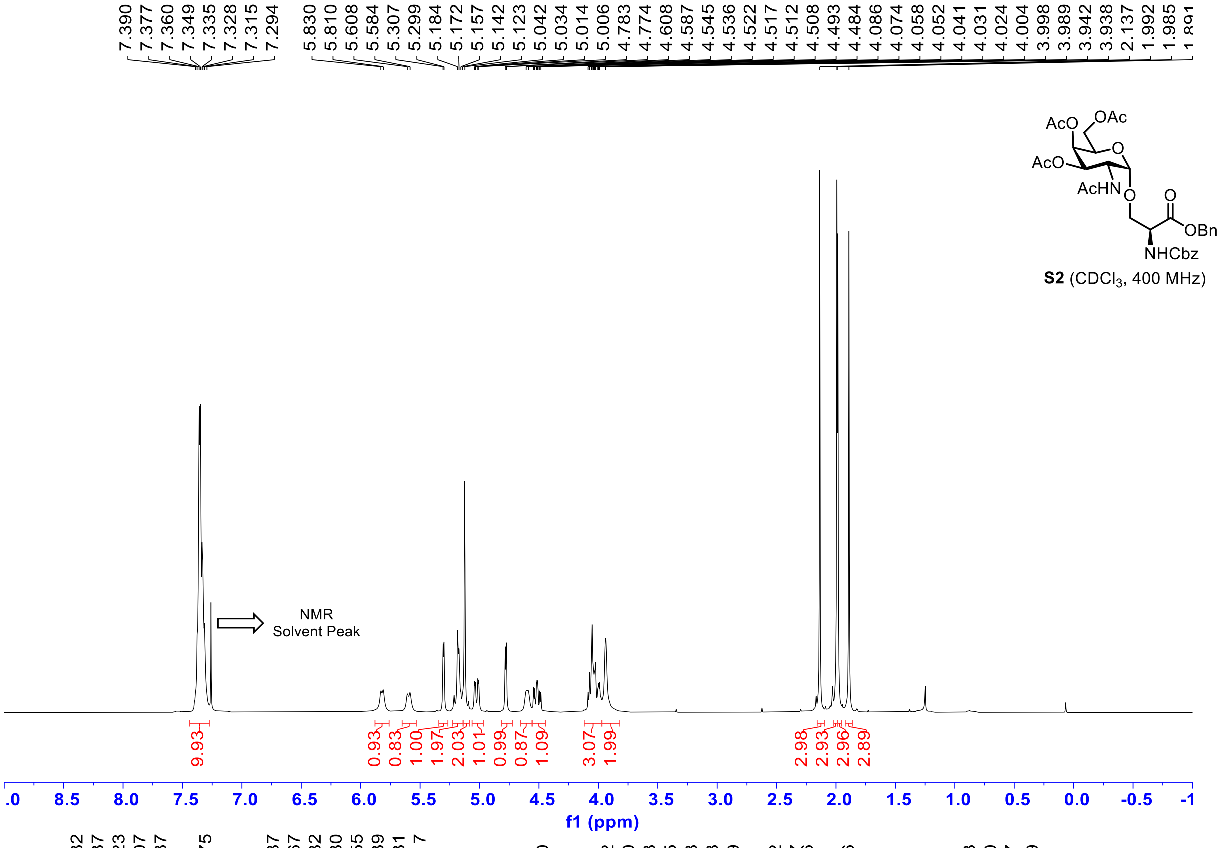

NMR Solvent Peak
S2 (CDCl$_3$, 400 MHz)
f1 (ppm)

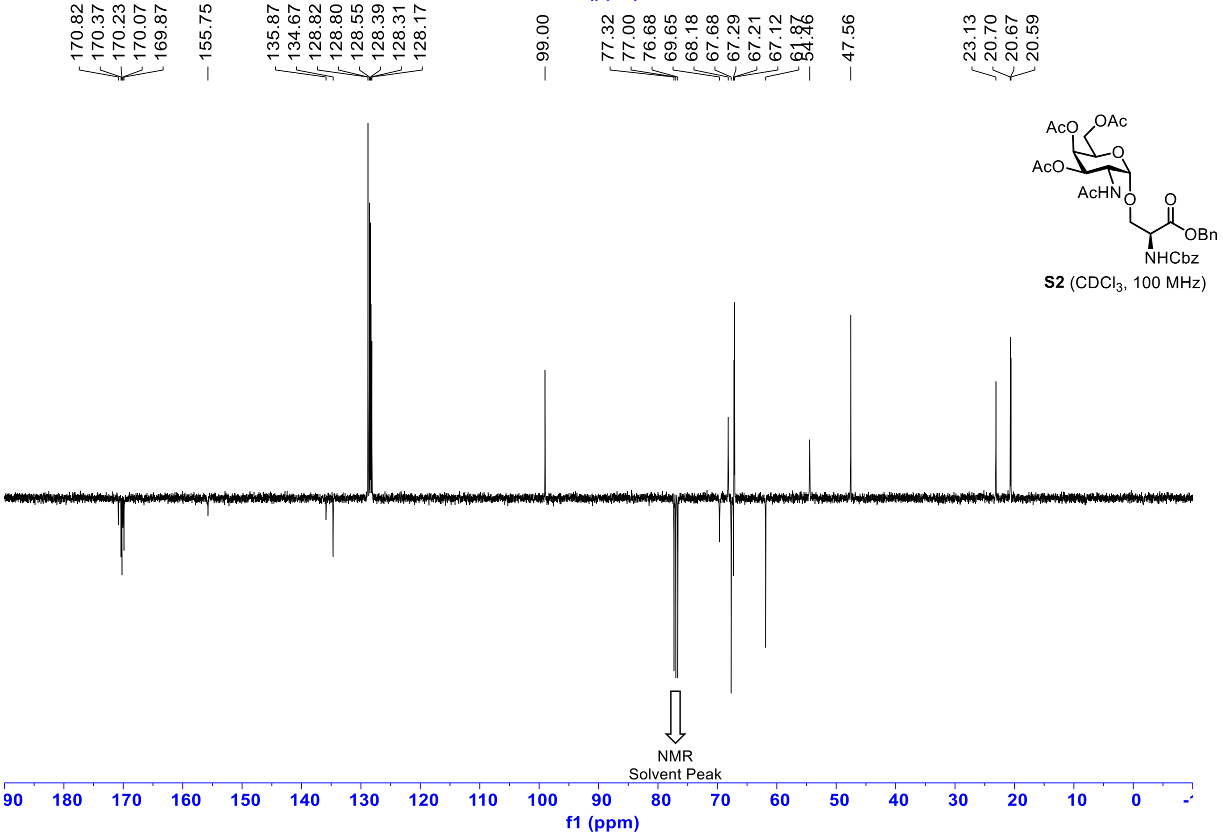

NMR Solvent Peak
S2 (CDCl$_3$, 100 MHz)
f1 (ppm)

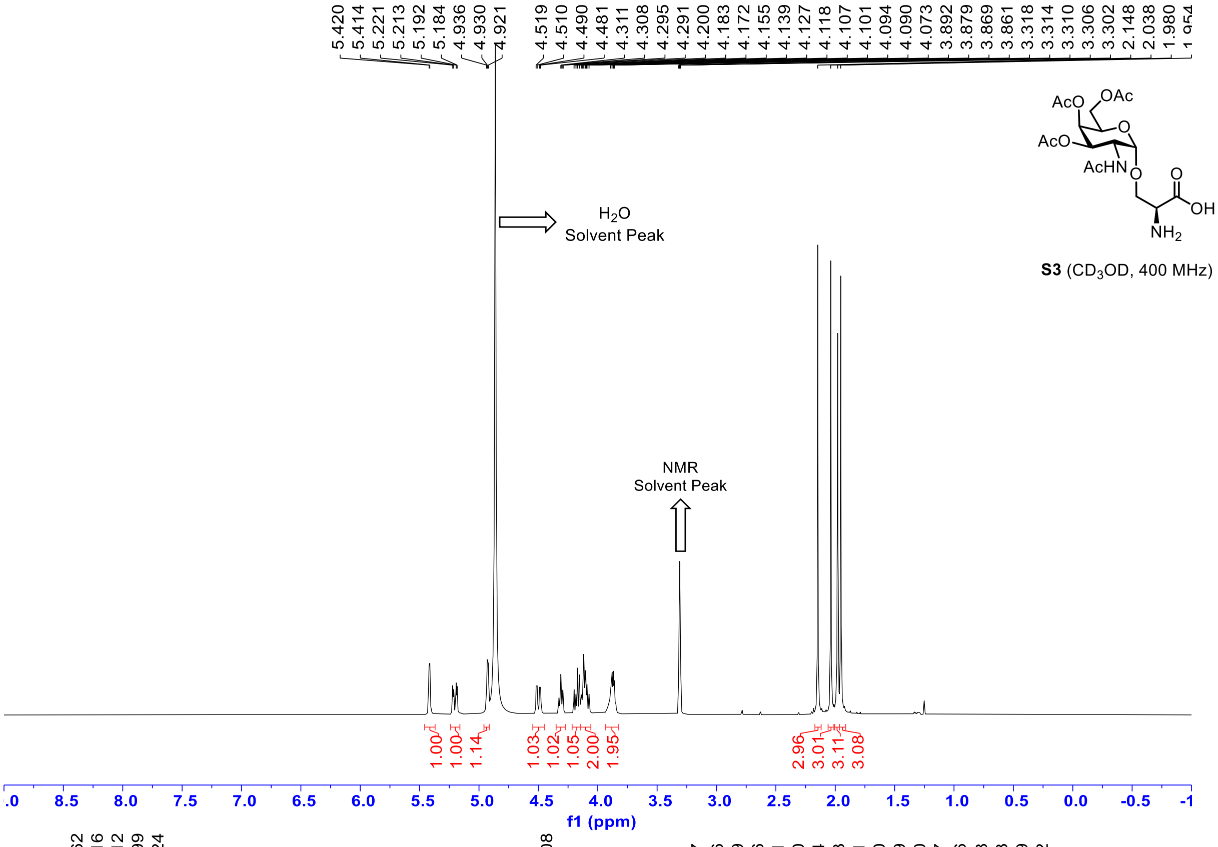

S3 (CD_3OD, 400 MHz)

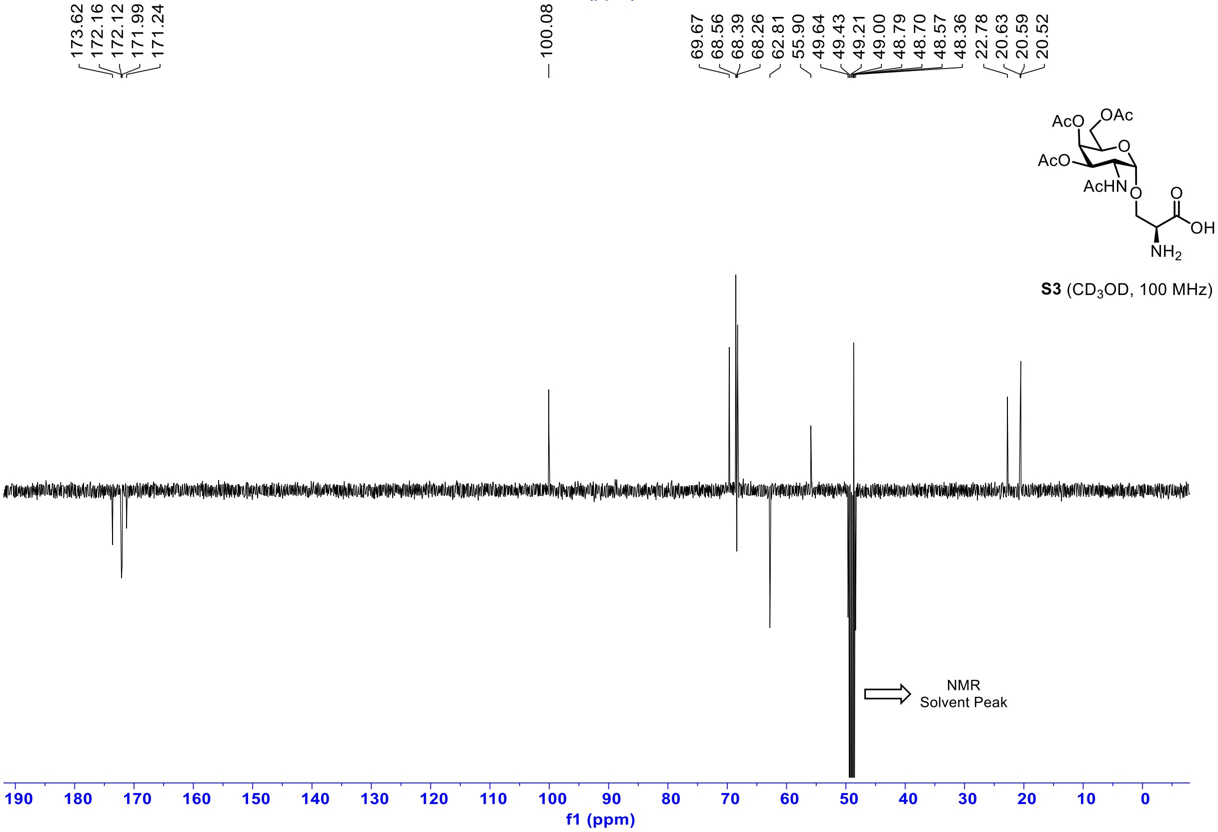

S3 (CD_3OD, 100 MHz)

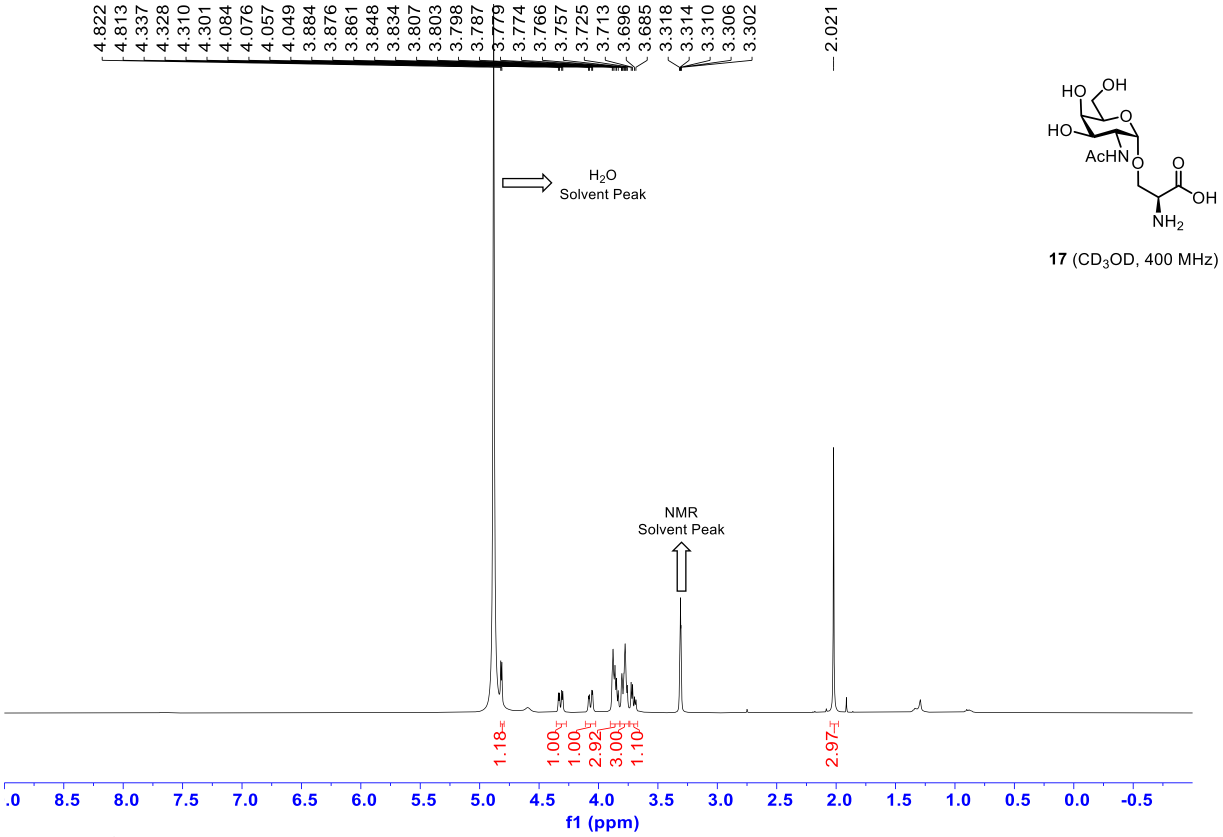
4.822
4.813
4.337
4.328
4.310
4.301
4.084
4.076
4.057
4.049
3.884
3.876
3.861
3.848
3.834
3.807
3.803
3.798
3.787
3.779
3.774
3.766
3.757
3.725
3.713
3.696
3.685
3.318
3.314
3.310
3.306
3.302
2.021
H_2O Solvent Peak
NMR Solvent Peak
1.18
1.00
1.00
2.92
3.00
1.10
2.97
f1 (ppm)
HO
OH
O
HO
AcHN
O
O
OH
NH_2
17 (CD_3OD, 400 MHz)

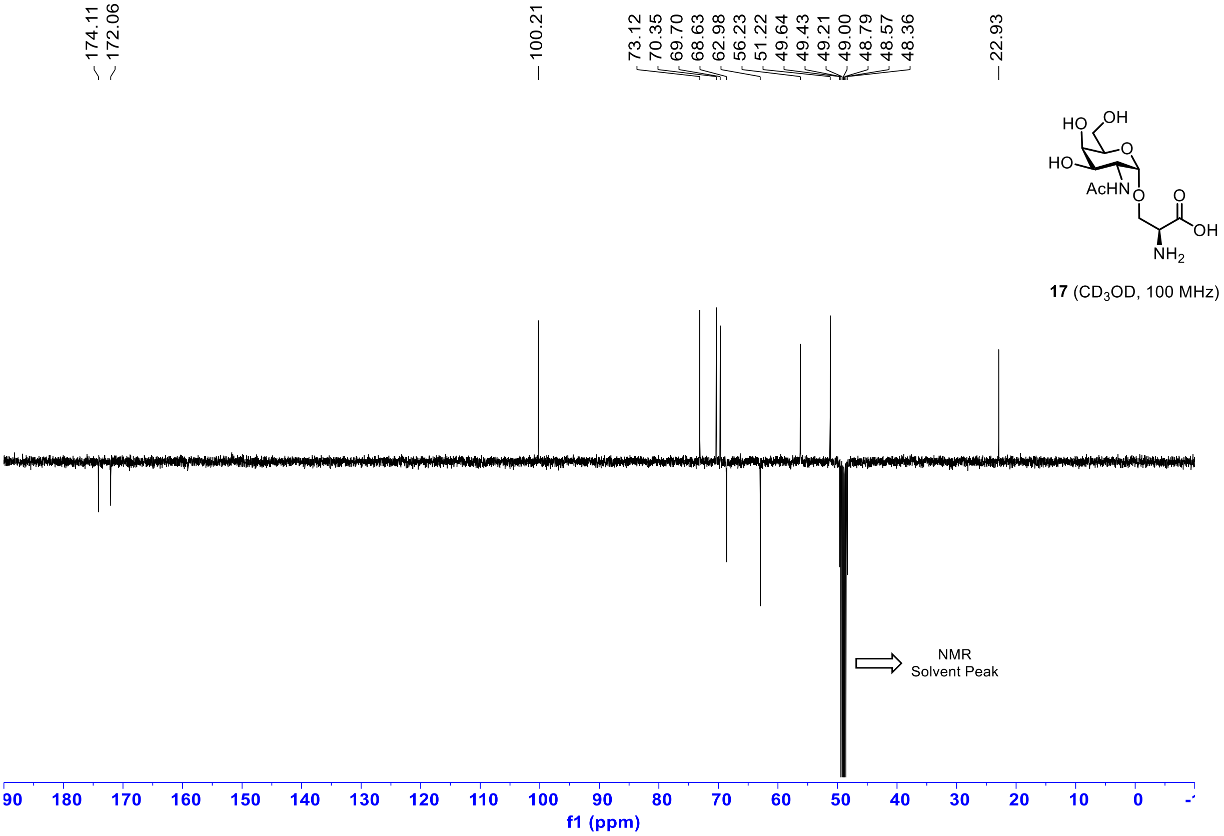
174.11
172.06
100.21
73.12
70.35
69.70
68.63
62.98
56.23
51.22
49.64
49.43
49.21
49.00
48.79
48.57
48.36
22.93
NMR Solvent Peak
f1 (ppm)
HO
OH
O
HO
AcHN
O
O
OH
NH_2
17 (CD_3OD, 100 MHz)
